# Design and development of non-magnetic hierarchical actuator powered by shape memory alloy based bipennate muscle

**DOI:** 10.1038/s41598-022-14848-w

**Published:** 2022-06-24

**Authors:** Kanhaiya Lal Chaurasiya, A. Sri Harsha, Yashaswi Sinha, Bishakh Bhattacharya

**Affiliations:** Department of Mechanical Engineering, Indian Institute of Technology Kanpur, Kanpur, 208016 India

**Keywords:** Aerospace engineering, Biomedical engineering, Mechanical engineering

## Abstract

Actuators are ubiquitous to generate controlled motion through the application of suitable excitation force or torque to perform various operations in manufacturing and industrial automation. The demands placed on faster, smaller, and efficient actuators drive innovation in actuator development. Shape memory alloy (SMA) based actuators have multiple advantages over conventional actuators, including high power-to-weight ratio. This paper integrates the advantages of pennate muscle of a biological system and the unique properties of SMA to develop SMA-based bipennate actuator. The present study explores and expands on the previous SMA actuators by developing the mathematical model of the new actuator based on the bipennate arrangement of the SMA wires and experimentally validating it. The new actuator is found to deliver at least five times higher actuation forces (up to 150 N) in comparison to the reported SMA-based actuators. The corresponding weight reduction is about 67%. The results from the sensitivity analysis of the mathematical model facilitates customization of the design parameters and understanding critical parameters. This study further introduces an Nth level hierarchical actuator that can be deployed for further amplification of actuation forces. The SMA-based bipennate muscle actuator has broad applications ranging from building automation controls to precise drug delivery systems.

## Introduction

**Figure 1:**
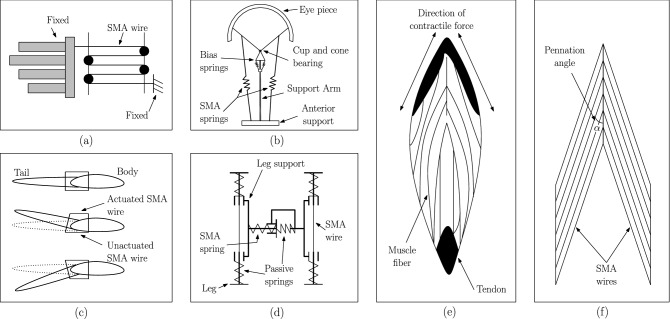
shows the schematic of the existing SMA based actuator design *vis-à-vis* bipennate muscle architecture, for instance (**a**) represents haptic force interaction, where hand-shaped device actuated using SMA wires is installed on a two-wheeled autonomous mobile robot^9,10^, (**b**) a robotic eye orbital prosthesis with an antagonistically mounted SMA spring-actuated eye orbital prosthesis. The position of the prosthetic eye is controlled by a signal from the ocular muscle of the eye^11^, (**c**) SMA actuators are ideal for underwater applications because to their high frequency response and low bandwidth. In this configuration, SMA actuators are used to create wave form motion by simulating the motion of a fish^10^, (**d**) SMA actuators are used to build micro robots for pipe inspection that can move inside a pipeline using the inch-worm motion principle actuated by SMA wires^10^, (**e**) shows the direction of contraction of muscle fibres and contractile force generation in a *gastrocnemius* pennate muscle tissue, (**f**) shows the arrangement of SMA wires in the shape of muscle fibres in the pennate muscular architecture.

Biological systems, such as the muscle architecture of mammals, can inspire a variety of nuanced actuators^1^. Mammals have a diverse set of muscle architecture, each serving a particular purpose. Nonetheless, most of the architecture in the mammalian musculature can be generalized into two vast categories; Parallel and Pennate. Found in the hamstring and other flexor muscles, as the name imp lies, parallel musculature has muscle fibers parallel to the central tendon. A succession of muscle fibers is placed in a row and functionally linked by the connective tissue surrounding them. Although these muscles are said to have a large excursion (shortening percentage), their total muscular force is quite limited. Contrastingly, pennate musculature is found in each muscle of the triceps surae complex^2^ (*gastrocnemius lateralis* (GL)^3^, *gastrocnemius medialis* (GM)^4^ and *soleus* (SOL)) and, on the extensor side of the thigh (*quadriceps femoris*)^5–7^. In Pennate architecture, the muscle fibers in bipennate muscle tissue are present on both sides of the central tendon at an oblique angle (angle of pennation). Pennate is derived from the Latin word ‘*penna*’, which means feather, accounting for its feather-like appearance as illustrated in Fig. 1. The fibers of the pennate muscles are shorter and oriented at an angle to the longitudinal axis of the muscles. Because of the pennation, the entire excursion of these muscles is reduced, resulting in a lateral and longitudinal component to the shortening process. On the other hand, because of the way physiological cross-sectional area is measured, activation of these muscles generate a higher total muscle force^8^. Thus, in a given cross-sectional area, a pennate muscle would be stronger and produce a higher force when compared to a parallel fiber muscle. The force produced in a single fiber causes macro-level muscle force generation in this musculature. Additionally, it has unique properties such as fast contraction, damage prevention during extension, and shock absorption. It transforms the relationship between fiber input and muscle force output by leveraging the unique characteristics and geometric complexity of fiber arrangement concerning the muscle line of action.

Actuators have become an essential component of mechanical systems due to their wide range of applications. As a result, the need for smaller, faster, and more effective actuators has become of paramount importance. Although conventional actuators have their advantages, their maintenance have proven to be costly and time-consuming. Hydraulic and pneumatic actuators are complex in their design and expensive, and are prone to wear, lubrication problems, and component failures. In response to the need, there is an emphasis on the development of cost-effective, size-optimized, and advanced actuators based on the smart materials. The current research looks towards shape memory alloy (SMA) based hierarchical actuators in order to meet this need. Hierarchical actuators are one-of-a-kind in that they combine a number of discrete actuator elements into geometrically complicated macro-scale subsystems to provide increased and expanded functionality. In this regard, the aforementioned human muscle tissue serves as a perfect multi-layered example for such hierarchical actuation. The current study describes a hierarchical SMA actuator with several individual actuator elements (SMA wires) arranged in the orientation of fibers present in a bipennate muscle, which improves the overall actuator performance.

The primary goal of an actuator is to generate mechanical output, such as force and displacement, by transforming electrical energy. Shape memory alloys are a class of smart materials that can restore their shape after being exposed to high temperatures. Under high loads, an increase in SMA wire temperature can cause shape recovery, resulting in high actuation energy density compared to various smart materials with direct coupling. Meanwhile when SMAs are subjected to mechanical stress, they become brittle. Under certain conditions, cyclical loading can absorb and discharge mechanical energy by demonstrating reversible hysteretic form change. These unique characteristics have made SMAs desirable for sensing, vibration damping, and especially for actuation applications^12^. Taking this into account, a significant amount of research has been conducted in the field of SMA-based actuators. It is imperative to note that SMA-based actuators have been designed to deliver both translational and rotational motion for variety of applications^13–15^. While some rotary actuators have been developed, researchers have been particularly interested in linear actuators. These linear actuators can be categorised into three types of actuators: one-dimensional, bias-force, and differential actuators^16^. Initially, hybrid actuators were created, combining SMA with other conventional actuators. One such example of a hybrid SMA-based linear actuator was the employment of SMA wires with a DC motor to provide a force output of approximately 100 N with a considerable displacement^17^.

One of the early developments in the domain of entirely SMA-based actuators was the parallel SMA actuator. Using numerous SMA wires, a parallel SMA-based actuator was designed to boost the force capability of actuator by placing all of the SMA wires in parallel^18^. Not only did the parallel connections of actuator necessitate more power, but the output force from a single wire was also limited. Another drawback of SMA-based actuators was the limited stroke they could achieve. To address this, a SMA wire actuated beam was created, which incorporated deflected flexible beams to magnify displacement and enable linear motion but failed to produce higher force^19^. Envisioned primarily for stroke amplification, a shape memory alloy-based soft morphing structure and robotic fabric were developed^20–22^. For areas that required large velocities, a compact actuator pump using thin-film SMA was reported for micro-pump actuator application^23^. The actuation frequency of thin film SMA membrane is the key factor controlling the speed of actuator. As a result, when compared to spring or strip-based SMA motors, wire-based SMA motors have better dynamic response. Soft robotics and gripper technology are two further applications in which SMA-based actuators have been used. For example, to replace the standard actuators used in the space gripper with a 25 N output force, a shape memory alloy-based parallel actuator was developed^24^. A SMA wire-based soft actuator with embedded matrix capable of generating a maximum pulling force of 30 N was produced in another scenario^25^. SMAs are also used to produce actuators by imitating biological phenomena due to their mechanical properties. One such development involved a 12 unit robot which was created by biomimicking an earthworm-like organism with SMA to generate a sinusoidal motion for actuation^26,27^.

As mentioned, there are limits to which the maximum force can be obtained from the existing SMA-based actuators. To address this challenge, the present study provides a bionic bipennate muscle architecture; driven by shape memory alloy wire. It envisages a hierarchical system that comprises a plurality of shape memory alloy wires. Hitherto, SMA based actuator involving similar architecture has not been reported in the literature. This unique and novel system based on SMA has been developed to explore the behavior of SMA in the bipennate muscle arrangement. The goal of this study was to create a bio-inspired bipennate actuator to generate significantly higher force in a small volume as compared to existing SMA-based actuators. The proposed SMA-based bipennate actuator design has been found to have a 67% lesser weight of the drive mechanism as compared to stepper motor-driven conventional actuators deployed in the HVAC building automation and controls domain. Henceforth, the terms muscle and actuator have been used interchangeably throughout this paper. The multiphysics modelling of such actuator was investigated in this study. The mechanical behaviour of such a system has been studied using both experimental and analytical methods. Under a 7V input voltage condition, the force and temperature distributions were further investigated. Subsequently, a parametric analysis was carried out to better understand the relationship between critical parameters and the output force. Finally, a hierarchical actuator has been envisaged, and the impact of the hierarchy level has been presented as a potential future scope in constructing non-magnetic actuators for prosthetic applications. According to the results of the aforementioned studies performed, a force at least four to five times that of the reported SMA-based actuators was generated using single-stage architecture. Furthermore, the same actuator force generated by multi-stage hierarchical actuators was shown to be more than ten times that of conventional SMA-based actuators. The study further reported the key parameters using sensitivity analysis amongst various design and input variables. The initial length of the SMA wire in each unipennate branch ($l_0$), angle of pennation ($\alpha$) and number of unipennate branches (*n*) had a strong and inverse impact on the magnitude of actuation force, while the input voltage (electrical energy) was found to be positively correlated.

## Results and discussion

The SMA wire exhibits the shape memory effect (SME) phenomenon that occurs in the nickel-titanium (Ni-Ti) alloy family. Generally, SMAs exhibit two temperature-dependent phases, the low and the high-temperature phases. Both the phases have unique properties due to the presence of different crystal structures. In the austenite phase (high-temperature phase), which is present above the transformation temperature, the material shows high strength and cannot be easily deformed under load. The behavior of the alloy is similar to stainless steel; thus, it has the ability to withstand higher stress upon actuation. Exploiting this characteristic behavior of Ni-Ti alloy, the SMA wires are arranged obliquely to form an actuator. The corresponding analytical model has been formulated to understand the fundamental mechanics of the temperature-driven behavior of the SMA under the influence of various parameters and different geometries. A good agreement between the experimental and analytical results was achieved.

**Figure 2:**
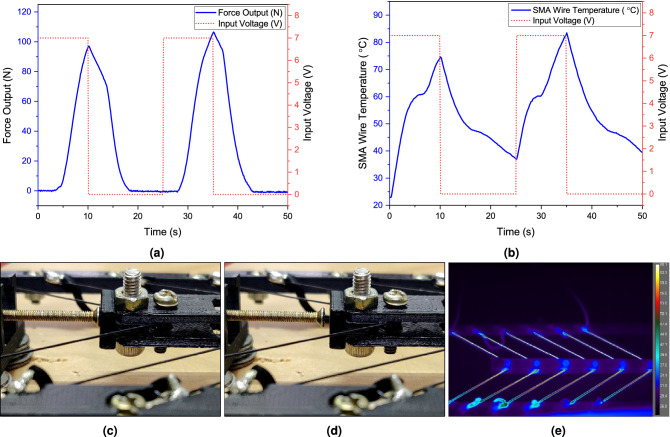
The plot depicts the experimental results for the temperature of the SMA wires as well as the force generated by the SMA-based bipennate actuator over two cycles. The input voltage was provided in two 10 s cycles (as shown by the red dots) with a 15 s cooling period between each cycle. The SMA wire used for the experimentation is the 0.51 mm diameter *Flexinol* wire from *Dynalloy, Inc.* (**a**) The graph depicts the experimental force obtained over the course of two cycles, (**c**, **d**) show two independent instances of the movable arm of actuator striking the PACEline CFT/5kN piezoelectric force transducer, (**b**) the graph depicts the maximum temperature across the entire length of the SMA wire during the two cycles, and (**e**) shows the snapshot of the temperature across the SMA wires obtained from the LWIR camera using the FLIR ResearchIR software. The geometric parameters considered for the experiment can be referred from Table 1.

The prototype depicted in Fig. 9a is experimentally investigated to assess the performance of the SMA-based bipennate actuator. Two of the characteristics, the force generated by the actuator (muscle force) and the temperature of the SMA wire (SMA temperature), have been experimentally measured. With a voltage differential established throughout the length of the wire in the actuator, the temperature of the wire was elevated through Joule heating effect. The input voltage was provided in two 10 s cycles (as shown by the red dots in Fig. 2a,b) with a 15 s cooling period between each cycle. A piezoelectric load cell was used to measure the blocked force while the temporal distribution of the temperature of the SMA wires was monitored in real-time using a high-resolution science-grade LWIR camera (refer Table 2 for specifications of both the equipment used). Figure 2b shows that during the high voltage phase, the temperature of the wire grows monotonically, but when no current is passed, the temperature of the wire declines consistently. In the present experimental setup, the temperature of SMA wires drops during the cooling stage; however, it remains above the ambient temperature. Figure 2e shows the snapshot of the temperature across the SMA wires obtained from the LWIR camera. Figure 2a on the other hand, depicts the block force generated by the actuator system. When the muscle force exceeds the restoring spring force, the movable arm, as illustrated in Fig. 9a, begins to move. As soon as the actuation begins, the movable arm comes in contact with the transducer, generating block force as shown in Fig. 2c,d. While the highest temperature was nearing $84\,^{\circ }\hbox {C}$, a maximum force of 105 N was observed.

The simulation outcome of the mathematical model and experimental results are compared for the 7V input voltage case as shown in the Fig. 5. Following the results obtained from the parametric analysis and avoiding the possibility of overheating the SMA wires, 11.2 W of power was supplied to the actuator. A programmable DC power supply was used to supply 7 V as the input voltage and 1.6 A of current was measured throughout the wire. The force generated by the actuator and the SMA temperature increased as the current was being supplied. In the case of the 7V input voltage condition, the maximum output force obtained in simulation results and experimental results for the first cycle are 78 N and 96 N, respectively. In the second cycle, the maximum output force was 150 N and 105 N for simulation and experimental results, respectively. The difference in the measurement of the blocked force and experimental data could be attributed to the blocked force measurement technique. The experimental result shown in Fig. 5a corresponds to the measurement of the blocked force, which in turn is measured when the actuator shaft comes in contact with the PACEline CFT/5kN piezoelectric force transducer as shown in Fig. 2c. Therefore, the force instantaneously becomes zero when the actuator shaft is not in contact with the force transducer at the start of the cooling zone as depicted in Fig. 2d. Furthermore, other parameters affecting the generation of force in subsequent cycles are the cooling time and convective heat transfer coefficient values in the preceding cycle. It is evident from Fig. 2b that with the 15 s cooling period, the SMA wire did not reach the room temperature and hence had a higher initial temperature ($40\,^{\circ }\hbox {C}$) during the second actuation cycle as compared to the first cycle ($25\,^{\circ }\hbox {C}$). Thus, the SMA wire temperature during the second heating cycle reaches the austenite start temperature ($A_s$) earlier when compared to the first cycle and stays in the transition period for a longer time, contributing to stress and force generation. On the other hand, the temperature profiles during heating and cooling cycles obtained from the experiment and simulation exhibit a high resemblance qualitatively to the instance of thermal imaging analysis.The comparative analysis of SMA wire thermal data from experiments and simulation has shown a good resemblance of fit during heating and cooling cycles and lies within the acceptable tolerance band of the experimental data. The maximum temperature of the SMA wire obtained in simulation and experimental results for the first cycle were $89\,^{\circ }\hbox {C}$ and $75\,^{\circ }\hbox {C}$, respectively, whereas, in the second cycle, the maximum temperature of the SMA wire was $94\,^{\circ }\hbox {C}$ and $83\,^{\circ }\hbox {C}$. The model developed in principle confirms the shape memory effect behavior. The role of fatigue and overheating were not taken into account in this investigation. In futuristic effort, the model will be refined to incorporate stress history of SMA wire to make it more suitable for engineering applications. The actuator output force and SMA temperature plots obtained from the Simulink block for 7V input voltage pulse condition lies within the acceptable tolerance band of the experimental data. This validates the correctness and robustness of the mathematical model developed.

### Simulation output

#### Case study for input pulse of 7 V

**Figure 3:**
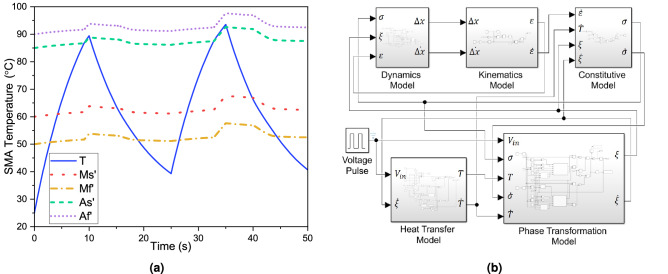
(**a**) Displays the simulation output of the temperature distribution as well as the stress-induced transition temperature of the SMA-based bipennate actuator. When the temperature of the wire crosses the austenite transition temperature in the heating phase, the modified austenite transition temperature starts to rise, and likewise the martensite transition temperatures drops when the temperature of the wire crosses the martensite transition temperature in the cooling phase, (**b**) a Simulink block diagram of the mathematical model for a bipennate-based SMA linear actuator that was utilised for analytical modelling of the actuation process. (Refer appendix section of the supplementary file for a detailed view of each subsystem of the Simulink model).

**Figure 4:**
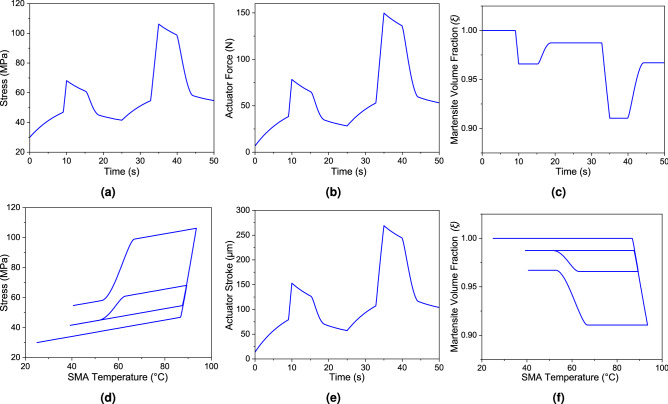
Under the input condition of 7 V for two cycles, analytical findings for the distribution of various parameters are displayed (10 s heating and 15 s cooling cycle). While (**a**–**c**) and (**e**) depicts a temporal distribution, on the other hand (**d**) and (**f**) illustrates distribution over temperature. For the concerned input condition, the maximum stress observed was 106 MPa (which was less than 345 MPa, the yield strength of the wire) , with a force of 150 N and a maximum displacement of 270 $\upmu$m, and the minimum martensite volume fraction was 0.91. On the other hand, the variation of stress and variation of martensite volume fraction with temperature resembles the hysteresis property.

A mathematical model has been developed in the MathWorks Simulink R2020b environment using the governing equations described in the methods section. Figure 3b shows the Simulink block diagram of the mathematical model. The model has been simulated for the input voltage pulse of 7V, as shown in Fig. 2a,b. The values of the parameters used in the simulation are listed in the Table 1. The transient results of the simulations are plotted in Figs. 3a and  4. Figure 4a,b show the stress induced in the SMA wire and the force generated by the actuator with respect to time. During reverse transformation (heating), when the SMA wire temperature, $T < A_s^{\prime}$ (stress-modified austenite phase start temperature), the rate of change of martensite volume fraction ($\dot{\xi }$) will be zero. Therefore, the rate of change of stress ($\dot{\sigma }$) will depend on the strain rate ($\dot{\epsilon }$) and the temperature gradient ($\dot{T}$) only, as governed by equation (1). However, as the SMA wire temperature increases and crosses ($A_s^{\prime}$), the austenite phase starts to form and the ($\dot{\xi }$) takes a value as given by equations (3). Hence, the stress change rate ($\dot{\sigma }$) is collectively governed by $\dot{\epsilon }, \dot{T}$ and $\dot{\xi }$ as given in equation (1). This explains the change in the gradient observed in the time-dependent stress and force plots during the heating cycle as shown in Fig. 4a,b.

**Table 1:** The list of parameters and values used for analytical modelling. The *A* and *M* subscripts in the parameters modulus of elasticity (*E*), slope (*C*), and resistivity (*r*) represent the austenite and martensite phase values, respectively. The *s* and *f* subscripts for the austenite and martensite transition temperatures denote the beginning and end of the transition temperature, respectively. Additionally, the material properties such as the density of the wire ($\rho$), specific heat of the wire ($c_p$), latent heat of the SMA ($\Delta H$), thermal expansion factor ($\theta _T$) have been listed in this table. Furthermore, the angle of pennation ($\alpha$), maximum transformation strain ($\epsilon _L$), diameter of the wire (*d*), initial length of the SMA wire in each unipennate branch ($l_0$), number of unipennate branches (*n*), and the bias spring constant ($K_x$) have been listed as values of parameters deployed in the design. As for the input conditions of the setup, initial spring deformation ($x_0$), convective heat transfer co-efficient ($h_T$), ambient temperature ($T_{\infty }$) and input voltage ($V_{in}$) have also been mentioned in the table.

Parameter	Value	Unit	Parameter	Value	Unit	Parameter	Value	Unit
$\alpha$	40	deg	$c_p$	836.8	J/kg-K	$C_A$	10	MPa/$^{\circ }$ C
$\epsilon _L$	0.04	-	$h_T$	70	W/m$^2$-K	$C_M$	10	MPa/$^{\circ }$ C
*d*	0.51	mm	$\Delta H$	24	kJ/kg	$A_{s}$	82.9	$^{\circ }$ C
$l_0$	83	mm	$T_{\infty }$	25	$^{\circ }$ C	$A_{f}$	87.5	$^{\circ }$ C
*n*	12	-	$\theta _T$	0.55	MPa/$^{\circ }$ C	$M_{s}$	57.9	$^{\circ }$ C
$x_0$	85	mm	$V_{in}$	7	V	$M_{f}$	47.5	$^{\circ }$ C
$K_x$	584	N/m	$E_A$	75	GPa	$r_A$	100	$\upmu \Omega$-cm
$\rho$	6450	kg/m$^3$	$E_M$	28	GPa	$r_M$	80	$\upmu \Omega$-cm

The same explanation holds true for the forward transformation (cooling) from austenite to martensite phase with the relation between the SMA wire temperature (*T*) and the stress-modified martensite phase finish temperature ($M_f^{\prime}$) as well. Figure 4d,f show the variation of the stress induced in SMA wire ($\sigma$) and the martensite volume fraction ($\xi$) with respect to change in the SMA wire temperature (*T*) for two actuation cycles. Figure 3a shows the time dependent SMA wire temperature variation with respect to the input voltage pulse. It can be observed from the plot that the wire temperature keeps on increasing as the heat supply is provided followed by convective cooling during zero voltage condition. During heating, when the SMA wire temperature (*T*) crosses the stress-modified austenite phase start temperature ($A_s^{\prime}$), the reverse transformation from martensite to austenite phase starts to occur. At this stage, the SMA wire contracts and the force is generated by the actuator. Likewise during cooling, when the SMA wire temperature (*T*) crosses the stress-modified martensite phase start temperature ($M_s^{\prime}$), the forward transformation from austenite to martensite phase begins, and the actuator force decreases.

**Figure 5:**
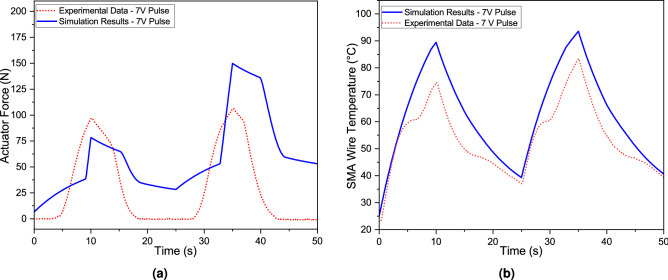
The comparison of the actuation parameters is obtained through experiments and analytical computations. The wires were subjected for 10 s with a 7V input pulse and then allowed to cool for 15 s (cooling phase) for two cycles. The pennation angle was set at $40^{\circ }$, and the initial length of the SMA wire in each unipennate branch was set at 83 mm. (**a**) The actuation force was measured using a load cell (**b**) Temperature of the wires were monitored using thermal IR camera.

The main qualitative aspects of the SMA-based bipennate actuator can be derived from the simulation results. In the case of voltage pulse input, the temperature of the SMA wire rises on account of Joule heating effect. The initial value of the martensite volume fraction ($\xi$) is set to one, as the material is initially in the complete martensite phase. As the wire is continuously heated, the temperature of the SMA wire goes beyond the stress-modified austenite phase start temperature $A_s^{\prime}$, resulting in the martensite volume fraction to decrease as shown in Fig. 4c. Additionally, Fig. 4e describes the temporal distribution of the stroke of the actuator and the actuation force variation with respect to time is shown in Fig. 5. The coupled set of equations involving temperature, martensite volume fraction, and stress developed in the wire results in the contraction of the SMA wire and the generation of force by the actuator. The variation of stress with temperature and variation of martensite volume fraction with temperature follows the hysteresis property of the SMA in the simulation case of 7V as shown in the Fig. 4d,f.

### Sensitivity analysis

**Figure 6:**
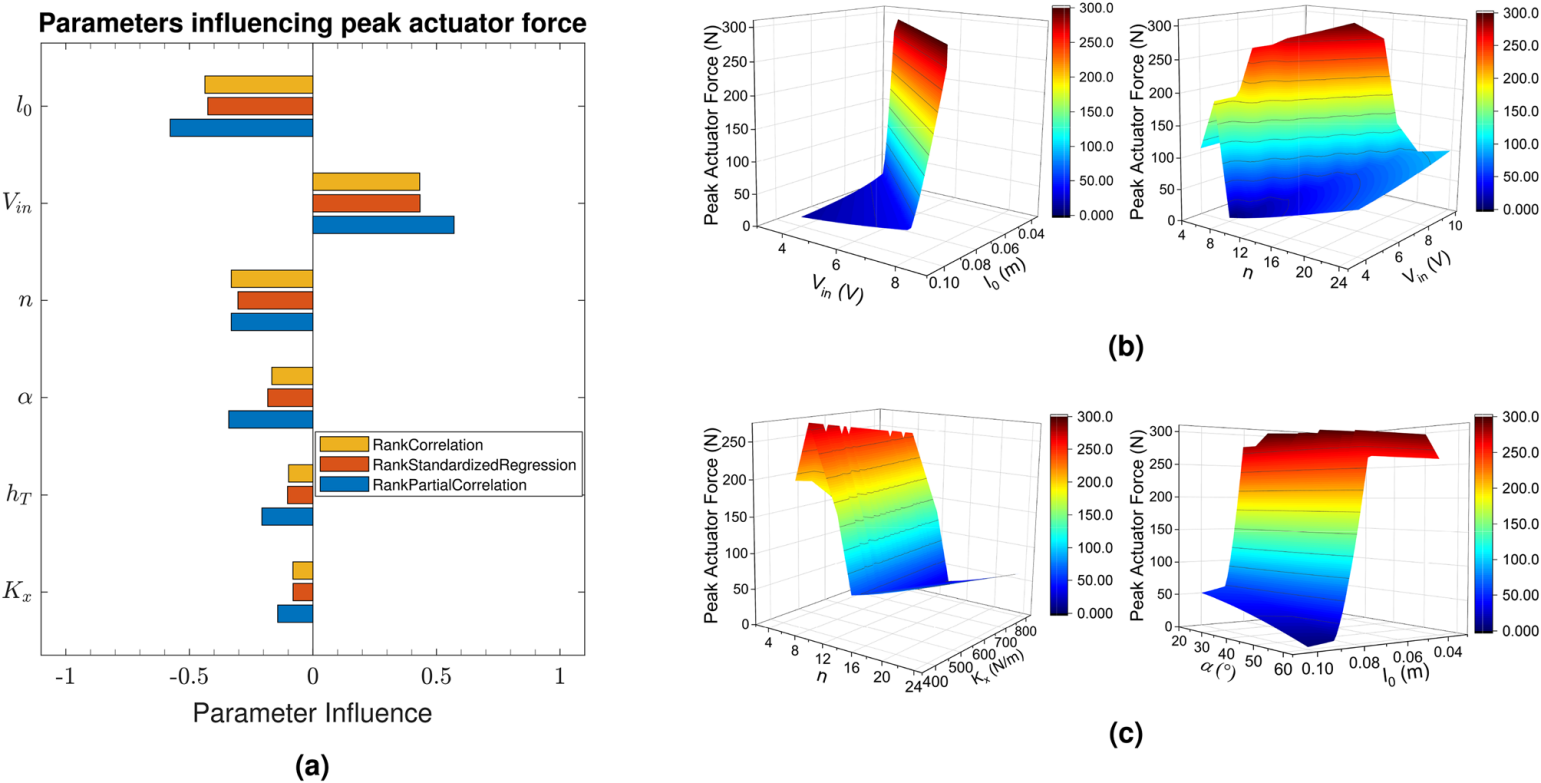
(**a**) The correlation coefficient values of the model parameters and their influence on the maximum output force for 2500 unique set for the mentioned model parameters are shown in a tornado plot. The graph depicts the rank correlation of several metrics. It is clear that $V_{in}$ is the sole positively correlated parameter, while $l_0$ is the most inversely correlated. The influence of various parameters in multiple combinations on the peak muscle force is depicted in (**b**, **c**). $K_x$ ranges from 400–800 N/m, while *n* ranges from 4 to 24. Voltage ($V_{in}$) has been altered from 4 to 10 V, wire length ($l_{0}$) has been varied from 40–100 mm and pennation angle ($\alpha$) has been varied from $20 - 60\,^{\circ }$.

In order to understand the influence of the physical parameters on the force output of the actuator, the sensitivity analysis study of the mathematical model has been carried out on the selected physical parameters to rank the parameters in the order of their influence. Firstly, the sampling of model parameters has been done using experimental design principles following uniform distribution (refer supplementary section on sensitivity analysis). In this case, the model parameters consist of input voltage ($V_{in}$), initial length of the SMA wire ($l_0$), pennation angle ($\alpha$), bias spring constant ($K_x$), convective heat transfer coefficient ($h_T$) and number of unipennate branches (*n*). In the next step, the peak muscle force has been chosen as the design requirement for the study and the parametric influence of each set of variables on the force generation is obtained. A tornado plot of the sensitivity analysis has been obtained in terms of correlation coefficients for each parameter, as shown in Figure 6a.

Figure 6a shows the tornado plot of the various correlation coefficients for each parameter against the design requirement of peak actuator force. It can be observed from Fig. 6a that the parameters, voltage ($V_{in}$) is directly correlated with the maximum output force, while the convective heat transfer coefficient ($h_T$), pennation angle ($\alpha$), bias spring constant ($K_x$) are inversely correlated with the output force and initial length of SMA wire ($l_0$) and number of unipennate branches (*n*) shows strong and inverse correlation. In case of direct correlation, the higher value of the correlation coefficient for the voltage ($V_{in}$) signifies that this parameter influences the force output the most. Another similar analysis is done to gauge the peak force by judging the influence of various parameters in multiple combinations of two design spaces, as shown in Fig. 6b,c. $V_{in}$ and $l_0$, $\alpha$ and $l_0$ have similar patterns with both the graph suggesting that higher value of $V_{in}$ and $\alpha$ with the lower value of $l_0$ resulting in higher peak force. The remaining two graphs are in agreement with Fig. 6a, with *n* and $K_x$ being negatively correlated while $V_{in}$ having positive correlation. This analysis helps in identifying and customizing the influential parameters through which the output force, stroke and efficiency of the actuator system can be tailored as per the requirement and application.

### Introducing the *N*th level hierarchical actuator

**Figure 7:**
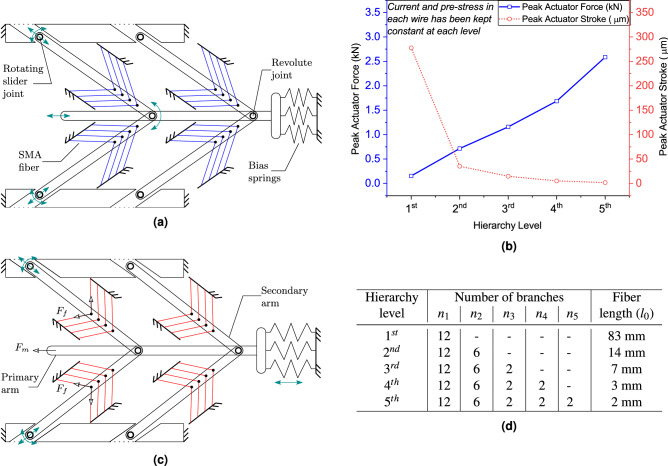
(**a**) A shape memory alloy-based two-stage ($N=2$) hierarchical linear actuation system is depicted in a bipennate configuration. The suggested model is implemented by replacing the SMA wires in the 1st level hierarchical actuator with another single stage hierarchical actuator. (**c**) depicts the deformed configuration of the 2nd level hierarchical actuator. (**b**) The distribution of force and displacement as a function of the number of hierarchy levels has been depicted. The peak actuator force has been found to be positively linked with hierarchy level in the graph, whereas the stroke has been shown to be inversely correlated with the hierarchy level. Throughout all the levels, the current and pre-stress in each wire were kept constant. (**d**) Table illustrates the number of branches in each level and the length of SMA wires (fibers). The wire characteristics are denoted by subscript 1, and the number of secondary arms (one attached to the primary arm) is denoted by the maximum number in subscript. For example, in level 5, $n_1$ refers to the number of SMA wires present in each bipennate structure, whereas $n_5$ refers to the number of secondary arms (one attached to the primary arm).

The current research work introduces and investigates a hierarchical actuator with *N* levels. In the two-stage hierarchy, as depicted in Fig. 7a, wherein instead of each SMA wire of the 1st level actuator, the bipennate arrangement has been implemented as shown in Fig. 9e. Figure 7c illustrates how the SMA wires are entwined with the movable arm (secondary arm) that moves only in its longitudinal direction. However, the primary movable arm continues to move in the same way as the movable arm of the 1st level hierarchical actuator. In general, the *N* level actuator is created by substituting the SMA wires of the $N-1$ level actuator with the first-level actuator. As a result, each branch mimics the first-level actuator, excluding the branch that holds the wire itself. In this manner, a nested structure can be formed, which generates a force multiple times to that generated by the first-level actuator. In the present study, a total of 1 m active length of SMA wire has been considered for each level, as shown in a tabular format in Fig. 7d. The current passed through each wire in each unipennate structure, along with the pre-stress as well as the stress developed in each SMA wire segment was same at each level. Based on our analytical model, output force was positively correlated with the level of the hierarchy, and the displacement was inversely correlated. Meanwhile, a trade-off between the displacement and muscle force was observed. As observed in Fig. 7b while the maximum force was obtained for the highest number of hierarchy levels, the maximum displacement was observed at the lowest level. A peak muscle force of 2.58 kN was detected when the hierarchy level was adjusted at $N=5$ wherein 2 $\upmu$m stroke was observed. On the other hand, the first-level actuator produced a force of 150 N with the stroke of 277 $\upmu$m. The hierarchical actuator manages to mimic an actual biological muscle, where the shape memory alloy based artificial muscle is able to generate a significant higher force with a precise and finer stroke. The limitation of this design for miniaturization is that with a higher hierarchy level, the stroke has been significantly reduced along with increasing complications during the fabrication of the actuator.

## Methods

### Governing equations

Various approaches have been proposed by numerous researchers in modeling the shape memory behavior of SMA which depends on thermo-mechanical properties accompanied by change in macroscopic crystal structure associated with the phase transformations. The formulation of constitutive law is complex in nature. The most commonly used phenomenological model was proposed by Tanaka^28^ which was extensively used for engineering applications. The phenomenological model proposed by Tanaka^28^ assumes the martensite volume fraction as an exponential function of temperature and stress. Later, Liang and Rogers^29^ and Brinson^30^ proposed a model in which phase transformation kinetics was assumed to be a cosine function of stress and temperature with few modifications to the model. A phase diagram based kinetic model was proposed by Bekker and Brinson^31^ to simulate the behavior of SMA material for arbitrary loading conditions, and also for partial transformation. Banerjee^32^ adopted phase diagram based kinetics approach of Bekker and Brinson^31^ to simulate the single degree of freedom manipulator developed by Elahinia and Ahmadian^33^. The phase diagram based kinetics approach which considers non-monotonic variation of stress with temperature is complex to implement for engineering applications. These shortcomings in the existing phenomenological models were addressed by Elahinia and Ahmadian^34^ and an enhanced phenomenological model was proposed to analyse and determine the shape memory behavior under any complex loading conditions.

#### Constitutive equation

The SMA wire constitutive model gives relationship between stress ($\sigma$), strain ($\epsilon$), temperature (*T*) and martensite volume fraction ($\xi$) of the SMA wire. The phenomenological constitutive model was first proposed by Tanaka^28^ and later adopted by Liang^29^ and Brinson^30^. The derivative form of the equation is given as follows:

1 $$\begin{aligned}&\dot{\sigma } = E\dot{\epsilon } + \theta _T \dot{T} + \Omega \dot{\xi } \end{aligned}$$

2 $$\begin{aligned} \dot{\sigma } = E(\dot{\epsilon } - \epsilon _L \dot{\xi }) + \theta _T \dot{T} \end{aligned}$$

where *E* is the phase-dependent Young’s modulus of SMA, is obtained using $\displaystyle E=\xi E_M + (1-\xi )E_A$ , $E_A$ and $E_M$ represents the Young’s modulus of austenite and martensite phase respectively, while the thermal expansion factor is represented by $\theta _T$. The phase transformation contribution factor is $\Omega = -E \epsilon _L$, and $\epsilon _L$ is the maximum recoverable strain in the SMA wire.

#### Phase transformation equations

The phase kinetics equations are the same cosine functions developed by Liang^29^ and later adopted by Brinson^30^ instead of the exponential function proposed by Tanaka^28^. The phase transformation model is an enhanced model presented by Elahinia and Ahmadian^34^ with modifications to phase transformation conditions given by Liang^29^ and Brinson^30^. The conditions used for this phase transformation model works effectively under complex thermo-mechanical loading. At every instant of time, the value of martensite volume fraction is calculated while simulating the constitutive equation.

- **Reverse transformation (Martensite to Austenite)**
  The governing equation of reverse transformation represented by the phase change from martensite to austenite during heating condition is given by:
  3 $$\begin{aligned} \xi = \frac{\xi _M}{2}\left[ \cos \left[ a_A \left( T - A_s \right) +b_A \sigma \right] + 1 \right] \end{aligned}$$
  where $\xi$ is the martensite volume fraction, $\xi _M$ is the martensite volume fraction reached before heating, $\displaystyle a_A = \pi /(A_f - A_s)$, $\displaystyle b_A = -a_A/C_A$ and $C_A$ are curve-fitting parameters, *T* is temperature of the SMA wire, $A_s$ and $A_f$ are austenite phase start and finish temperatures, respectively.
- **Forward transformation (Austenite to Martensite)**
  The governing equation of forward transformation represented by the phase change from austenite to martensite during cooling condition is given by:
  4 $$\begin{aligned} \xi = \frac{ 1- \xi _{A}}{2} \cos \left[ a_M \left( T - M_f\right) + b_M \sigma \right] + \frac{1+\xi _{A}}{2} \end{aligned}$$
  where $\xi _A$ is the martensite volume fraction reached before cooling, $\displaystyle a_M = \pi /(M_s - M_f)$, $\displaystyle b_M = -a_M/C_M$ and $C_M$ are curve-fitting parameters, *T* is temperature of the SMA wire, $M_s$ and $M_f$ are martensite start and finish temperatures, respectively.

Upon differentiating equations (3) and (4), the reverse and forward transformation equations reduce to the form as follows:

5 $$\begin{aligned} \dot{\xi } = \eta _{\sigma } \dot{\sigma } + \eta _{T} \dot{T} \end{aligned}$$

During the forward and reverse transformations, $\eta _{\sigma }$ and $\eta _{T}$ assume different values. The governing equations related to $\eta _{\sigma }$ and $\eta _{T}$ have been derived and discussed in detail in the supplementary section.

#### Heat transfer equation

The heat energy required to increase the SMA wire temperature is obtained through the Joule heating effect. The heat energy absorbed or released in SMA wire is represented by the latent heat of transformation. The heat loss in the SMA wire is due to forced convection and considering the radiation effects to be negligible, the heat energy balance equation is given as follows:

6 $$\begin{aligned} {m_{{ wire}}c_p}\dot{T} = \frac{V_{in}^2}{R_{{ ohm}}} - A_{{ c}}h_T(T-T_{\infty }) + m_{{ wire}}\Delta H \dot{\xi } \end{aligned}$$

where $m_{wire}$ is the total mass of SMA wire, $c_{p}$ is the specific heat capacity of the SMA, $V_{in}$ is the applied voltage across the wire, $R_{ohm}$ is the phase-dependent resistance of SMA given by; $R_{ohm} = (l/A_{cross})[\xi r_M + (1-\xi )r_A]$ with $r_M$ and $r_A$ as resistivity of SMA in martensite and austenite phases, respectively, $A_{c}$ is the curved surface area of the SMA wire, $\Delta H$ is the latent heat of transformation of the shape memory alloy wires, *T* and $T_{\infty }$ represent the temperature values of the SMA wire and ambient environment respectively.

#### Bipennate muscle stiffness equation

When the shape memory alloy wires are actuated, the wires contract producing a force in each branch of the bipennate arrangement, known as the fiber force. The fiber force in each branch of SMA wire collectively generates a muscle force for actuation, as shown in the Fig. 9e. Due to the presence of bias spring, the total muscle force of the *N*th level hierarchical actuator is given as follows:

7 $$\begin{aligned} F_m = \prod _{i=1}^{n=N} [n_{i}F_f\cos (\alpha _i)] -K_x \left( \Delta x + x_0\right) \end{aligned}$$

Substituting, $N = 1$, in equation (7) the muscle force for a 1st level bipennate actuator prototype can be obtained as:

8 $$\begin{aligned} F_m = n F_f \cos \alpha - K_x\left( \Delta x+x_0\right) \end{aligned}$$

where *n* is the number of unipennate branches, $F_m$ is the muscle force generated by the actuator, $F_f$ is the fiber force in the SMA wire, $K_x$ is the stiffness of the bias spring, $\alpha$ is the angle of pennation, $x_0$ is the initial displacement of the bias spring to maintain the SMA wires in the pre-tension arrangement, $\Delta x$ is the stroke of the actuator.

The total displacement or stroke ($\Delta x$) of the actuator as a function of stress induced ($\sigma$) and strain developed ($\epsilon$) in the SMA wire for a *N*th level actuator is given by (refer supplementary section for derivation):

9 $$\begin{aligned} \Delta x_N= \frac{\prod _{i=1}^{n=N} [n_{i}F_f\cos \alpha _i ] -K_x x_0}{\prod _{i=1}^{n=N} [n_{i}] A_{cross} \left[ -\frac{E}{l_{10}}\cos ^2\alpha _1 +\sum _{k=2}^{n=N}\left( \frac{\sigma }{l_k} \sin ^2\alpha _k \prod _{j=1}^{k-1} \cos \alpha _j \right) +\frac{\sigma }{l_{10} (1-\epsilon )} \sin ^2 \alpha _1 \right] } \end{aligned}$$

Substituting, $N=1$, in equation (9), the stroke for a bipennate actuator is given as:

10 $$\begin{aligned} \Delta x_{1} = \frac{l_0(1-\epsilon )\left[ n \sigma A_{cross} \cos \alpha - K_{x} x_0\right] }{n A_{cross} \left[ \sigma \sin ^2 \alpha -E (1-\epsilon )\cos ^{2} \alpha \right] } \end{aligned}$$

#### Kinematics equation

**Figure 8:**
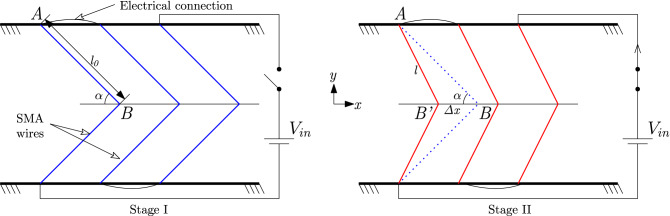
All the *n* unipennate structure ($n=6$ in this figure) have been connected in series with $V_{in}$ as the input voltage. Stage I: schematic of SMA wires in a bipennate configuration under zero voltage condition; Stage II: depicts the structure under actuation where the SMA wires have contracted because of the reverses transformation as represented by red lines.

The kinematics equation gives the relation between strain ($\epsilon$) and displacement or stroke ($\Delta x$) of the actuator. The strain in the SMA wire as a function of the initial length of the SMA wire ($l_0$), and the length of the wire (*l* ) at any time *t* in a single unipennate branch is given as follows:

11 $$\begin{aligned} \epsilon = \frac{l_0-l}{l_0} \end{aligned}$$

where $l = \sqrt{l_0^2 +(\Delta x_1)^2 - 2 l_0 (\Delta x_1) \cos \alpha _1}$ is obtained by applying cosine formula in $\Delta$ABB’, as shown in Fig. 8. For the 1st level actuator ($N = 1$), the variables $\Delta x_1$ is the $\Delta x$ and $\alpha _1$ is the $\alpha$ as depicted in Fig. 8, Upon differentiating equation (11) with respect to time, and substituting the value of *l*, the strain rate can be written as follows:

12 $$\begin{aligned} \dot{\epsilon } = \frac{(\Delta \dot{x_1}) \cos \alpha }{ \sqrt{l_0^2 - 2l_0 (\Delta x_1)\cos \alpha }} \end{aligned}$$

Generalizing the strain rate for *N*th level actuator , the equation modifies as follows:

13 $$\begin{aligned} \dot{\epsilon } = \frac{(\Delta \dot{x_N}) \prod _{j=1}^{N} \cos \alpha _j}{ \sqrt{l_0^2 - 2l_0 (\Delta x_N) \prod _{j=1}^{N} \cos \alpha _j}} \end{aligned}$$

where $l_0$ is the initial length of the SMA wire, *l* is the length of the wire at any time *t* in a single unipennate branch and $\epsilon$ is the strain developed in the SMA wire, $\alpha$ is the pennation angle and $\Delta x$ is the displacement of the actuator (as illustrated in Fig. 8) .

### Concept and prototyping

**Figure 9:**
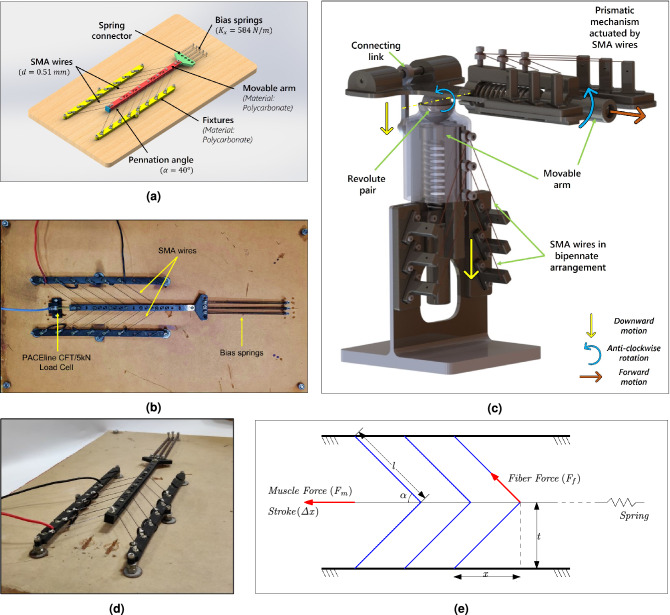
Proposed SMA-based linear actuation system with a bipennate configuration, where the SMA wires have been arranged obliquely. (**a**) depicts the CAD model of the prototype with some of the components mentioned along with their values used for the prototype, (**b**, **d**) represents the developed proof-of-concept prototype^35^. While (**b**) illustrates the top view of the prototype with electrical connection and bias springs along with the load cell used whereas, (**d**) shows the perspective view of the setup. (**e**) Schematic of the linear actuation system with SMA wires in a bipennate arrangement at any time *t*, showing the direction of fiber and muscle forces as well as the stroke. (**c**) A 2-DOF revolute-prismatic joint has been proposed deploying bi-planar SMA-based actuators. As shown in the diagram, the connecting link transfers linear motion from the lower actuator to the upper arm, resulting in a revolute joint. The motion of the prismatic pair, on the other hand, is identical to that of the 1st level hierarchical actuator.

As a proof of concept, a SMA-based bipennate actuator is developed to validate the simulation output of the governing equations with the experimental results. The CAD model of the bipennate linear actuator has been shown in Fig. 9a. On the other hand, Fig. 9c depicts a novel design proposed for a revolute-prismatic joint using bi-planar SMA-based actuator with bipennate architecture. The actuator components were fabricated using additive manufacturing with the help of Ultimaker 3 Extended 3D printer. The material used for the 3D printing of the components is poly-carbonate as it is tough, strong and has a high glass transition temperature (110–113 $^{\circ }$ C), making it suitable for a heat-resistant material. Furthermore, the shape memory alloy wires used for the experimentation are *Flexinol* actuator wires from *Dynalloy, Inc*, and the material properties corresponding to *Flexinol* wires are used in the simulation. The plurality of SMA wire is arranged in the form of fibers present in a bipennate muscle arrangement to obtain high force produced by the hierarchical actuator, as shown in Fig. 9b,d.

The acute angle made by the SMA wires with movable arm is termed as pennation angle ($\alpha$) as shown in Fig. 9a. The SMA wires were maintained at the required bipennate angle with the help of terminal crimps connected to the left and the right fixtures. The bias spring arrangement which is held to a spring connector is designed such that different sets of elongation of bias spring can be adjusted depending on the number (*n*) of SMA fibers. Furthermore, the arrangement of movable sub-assembly is designed in such a way that the SMA wires are exposed to outside environment for cooling under the action of forced convection. The top and bottom plate of movable sub-assembly can aid for cooling SMA wires through the extruded cuts designed for weight reduction. Furthermore, the two ends of SMA wire are fixed to the left and right fixtures respectively with the help of terminal crimps. A plunger is fastened to one end of movable sub-assembly to maintain spacing between top and bottom plates. The plunger also serves the purpose of exerting the blocked force on the transducer through contact to measure the block force when the SMA wires are actuated.

The bipennate SMA muscle architecture is connected electrically in series which is supplied with input pulsed voltage. In one cycle of voltage pulse, when voltage is supplied and the SMA wires are heated above austenite start temperature, the length of the wire in each branch contracts. This contraction results in the actuation of the movable arm sub-assembly. When the voltage is set to zero in the same cycle, the heated SMA wire is cooled down below martensite finish temperature, leading to the restoration to the original position. Under zero voltage condition, the SMA wires are initially extended passively with the help of bias spring to reach detwinned martensite state. With the contraction due to heating by passing voltage pulse to the SMA wires (the SMA reaches austenite phase), the screws through which SMA wires are wound moves resulting in the actuation of movable arm. When the SMA wires contract, the bias spring arrangement generates a force in the reverse direction by further elongation of the spring. When the voltage becomes zero in the pulsed voltage, the SMA wires elongate on cooling by forced convection and changes its shape reaching twinned martensite phase.

### Actuator force experiment

**Figure 10:**
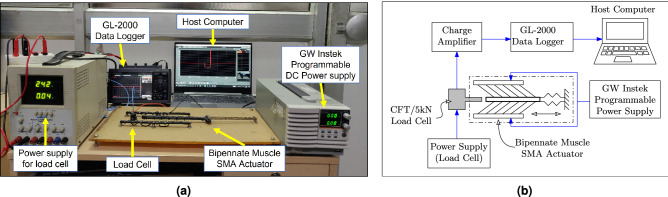
(**a**) The SMA-based linear actuation system was set up in an experiment to measure the force generated by the actuator. A load cell measures the block force and is powered by a 24V constant power supply. A 7 V voltage difference is applied throughout the full length of the cable using a GW Instek programmable DC power source. The SMA wire contracts as a result of the heating, and the movable arm comes in contact with the load cell exerting blocked force. The load cell is connected to the GL-2000 data logger, and the data is saved on the host computer for post processing. (**b**) A schematic showing the circuit of the components of the muscle force measurement experimental setup.

The prototype depicted in Fig. 9b is experimentally investigated to assess the performance of the SMA-based bipennate actuator. The experimental setup, as shown in Fig. 10a, consists of a programmable DC power supply to provide input voltage to the SMA wires. As illustrated in Fig. 10b, a piezoelectric load cell (PACEline CFT/5kN) is used to measure the blocked force using a Graphtec GL-2000 data logger. The data is recorded by a host computer for further investigations. A constant power supply is required for the load cell and charge amplifier to obtain the voltage signal. The corresponding signal is converted to force output based on the sensitivity and other parameters of the piezoelectric force transducer as mentioned in Table 2. The SMA wire temperature increases when a voltage pulse is applied, resulting in the contraction of the SMA wire, leading to the force generation of the actuator. The experimental result of the muscle force output for 7V input voltage pulse is shown in the Fig. 2a.

**Table 2:** Technical parameters of PACEline CFT/5kN piezoelectric force transducer and FLIR A655sc high-resolution science grade LWIR camera.

PACEline CFT/5kN load cell		FLIR A655sc LWIR camera	
Parameters	Value	Parameters	Value
Sensitivity	− 8.097 pC/N	Resolution	$640 \times 480$
Nominal (rated) force	5 kN	Field of view	$25^{\circ }\times 19^{\circ }$ ($31^{\circ }\hbox {diagonal}$)
Natural frequency	40 kHz	Frame rate	50 Hz
Output span	± 10 V	Lens type	IR Lens, $f = 24.6\ \hbox {mm}$
Breaking force	10 kN	Spectral range	7.5–14.0 $\upmu \hbox {m}$

### Thermal imaging experiment

**Figure 11:**
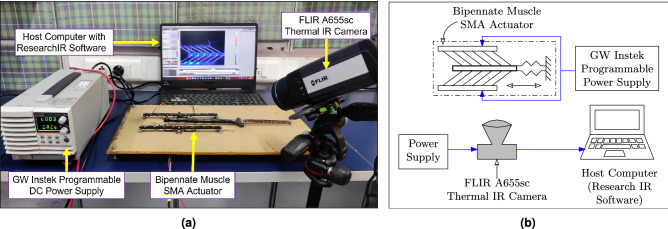
(**a**) The SMA-based linear actuation system was set up in an experiment to monitor the temperature of the SMA wire of the actuator. A 7 V voltage difference is applied throughout the full length of the cable using a GW Instek programmable DC power source. The actuator is kept in the focal plane of the FLIR A655sc Thermal IR camera to precisely monitor the closely packed SMA wires. (**b**) A schematic showing the circuit of the components of the thermal imaging experimental setup.

Shape memory alloy is stimulated with heat energy and thus temperature becomes an important parameter to study the shape memory effect phenomenon. Experimentally, the thermal imaging and temperature measurement of the SMA-based bipennate actuator prototype has been conducted as illustrated in Fig. 11a. A programmable DC power source provides an input voltage to the SMA wires in the experimental arrangement, as shown in Fig. 11b. The temperature variation of the SMA wires is measured in real-time using a high-resolution science-grade LWIR camera (FLIR A655sc). A host computer records the data using ResearchIR software for further post-processing. When a voltage pulse is applied, the temperature of the SMA wire rises, causing the SMA wire to contract. Figure 2b depicts the experimental result of time-dependent SMA wire temperature for a 7V input voltage pulse.

## Conclusion

This paper presented an innovative shape memory alloy-based hierarchical actuator that comprises SMA wires arranged in the form of fibers present in bipennate muscle tissue. The biological advantage of using pennate muscle is that the fibers present are obliquely inclined to the muscle line of action, allowing the fiber force to be coupled to macro-level muscle force, leading to higher force production. Furthermore, the stiffness of the hierarchical actuator is collectively governed by the change in SMA wire length, pennation angle, number of branches, stress developed attributing for variable stiffness characteristics. A mathematical model was developed for the SMA-based bipennate actuator along with a competent Simulink model to solve the set of implicit governing equations of the actuator. Mathematical modeling was followed by prototype development. The actuator prototype was fabricated and the experiments were conducted to measure the force generated by the actuator. Experimental data were consistent with the simulation results, thus validating the effectiveness of the mathematical model in defining the physics of the actuator and estimating the force generated by the system.

The current research work is partially motivated by the increasing demand from the actuator industry and presented a novel driving principle other than electro-magnetism providing an alternative for conventional actuators integrated with gear mechanisms. The bipennate muscle-based shape memory alloy actuator has broad applications ranging from building automation controls to precise drug delivery methods. The present invention also caters to the need for actuators in the study involving magnetic resonance imaging as the imaging is very susceptible to electromagnetic noise generated by conventional coil-based motors. Furthermore, this actuator can also be manifested into a two degree of freedom revolute-prismatic joint, which can be used in robotic manipulators and related applications. The developed system can also act as variable stiffness hierarchical actuator. The use of the proposed product will foster higher utilization rates for a broad-band of gripping force and hence a better cost-benefit ratio. As a part of future development, different envelope packaging and controller circuit will be designed and implemented to obtain size and power optimization. The size of the actuator can be further optimized by reducing the gap between two consecutive branches or by arranging the bipennate architecture into vertical stacks. These improvements will enable a higher output force from a compact size actuator. The current bio-mimic approach can also be used to develop a rotary motion for medium to high torque applications as well as a bio-inspired variable force gripper system with potential application in mobile robotics.

## Supplementary Information

Supplementary Information.

## Data Availability

All relevant data are available from the corresponding author upon reasonable request, and/or are included within the main article and supplementary information.
