# Supplementary material for: Design and development of non-magnetic hierarchical actuator powered by shape memory alloy based bipennate muscle
Source: Sci Rep. 2022 Jun 24;12:10758. doi: 10.1038/s41598-022-14848-w (PMC9232532; doi:10.1038/s41598-022-14848-w)
Supplement: Supplementary file 1 — Supplementary Information. [file 41598_2022_14848_MOESM1_ESM.pdf]

Supplementary Information:
Design and development of non-magnetic hierarchical actuator powered by shape memory alloy based bipennate muscle

Kanhaiya Lal Chaurasiya[1], A Sri Harsha[1], Yashaswi Sinha[1], and Bishakh Bhattacharya[1*]

[1]Department of Mechanical Engineering, Indian Institute of Technology Kanpur, Kanpur, 208016, India
[*]Corresponding author: bishakh@iitk.ac.in

Contents

1 Supplementary Introduction **1**
1.1 Shape Memory Alloys . . . 1
1.2 Market access information . . . 2
1.3 Comparison with existing SMA-based actuators . . . 2
2 Supplementary Results and Discussion **3**
2.1 Case study for variable input voltage pulse . . . 3
2.2 Sensitivity analysis . . . 4
2.3 Effect of input voltage and forced convection on actuation frequency . . . 6
3 Supplementary Methods **8**
3.1 Phase transformation equations . . . 8
Reverse transformation (Martensite to Austenite) • Forward transformation (Austenite to Martensite)
3.2 Bipennate muscle stiffness equation – **1st** hierarchical level actuator . . . 9
3.3 Bipennate muscle stiffness equation – **2nd** hierarchical level actuator . . . 10
3.4 Bipennate muscle stiffness equation – **3rd** hierarchical level actuator . . . 12
3.5 Bipennate muscle stiffness equation – **Nth** hierarchical level actuator . . . 15
4 Application **15**
5 References **15**
6 Notation **16**
7 Appendix: Simulink block subsystems **17**
7.1 Constitutive Model: Simulink subsystem . . . 17
7.2 Heat Transfer Model: Simulink subsystem . . . 17
7.3 Phase Transformation Model: Simulink subsystem . . . 18
Heating Equations: Simulink sub-model • Heating Conditions: Simulink sub-model • Cooling Equations: Simulink sub-model • Cooling Conditions: Simulink sub-model
7.4 Dynamics Model: Simulink subsystem . . . 20
7.5 Kinematics Model: Simulink subsystem . . . 20

1 Supplementary Introduction

1.1 Shape Memory Alloys

Shape Memory Alloys (SMAs) are a distinct set of smart materials capable of recovering their shape at an elevated temperature phase. The increase in SMA wire temperature under high loads can lead to shape recovery, which results in high actuation energy densities compared to different smart materials that exhibit direct coupling. When SMAs are subjected to mechanical cyclic loading, SMAs can absorb and dissipate mechanical energy by exhibiting reversible hysteretic shape change under specific conditions. These unique characteristics have made SMAs desirable for sensing, vibration damping, and especially for actuation applications[1].

The SMA wire exhibits the shape-memory phenomenon that occurs in the nickel-titanium alloy family. The concentration of the elements is a critical variable in an alloy comprising two or more elements. The presence of nickel and titanium atoms in the alloy is almost in equal ratio 1:1, resulting in the formation of a crystal structure that can transform from one form to another. The temperature at which such phase transformation occurs depends upon the exact composition of the alloy. In the austenite phase, which is present above the transformation temperature, the material shows high strength and cannot be easily deformed under load. The behavior of the alloy is similar to stainless steel; thus, it has the ability to withstand higher stress upon actuation. Generally, SMAs exhibit two temperature-dependent phases, the low and the high-temperature phases. Both the phases have unique properties due to the presence of different crystal structures. The low-temperature phase known as martensite (M) exhibits monoclinic, orthorhombic, or tetragonal crystal arrangements. On the other hand, the high-temperature phase called austenite (A) has a cubic crystal structure. The shear lattice distortion results in the transformation from one phase to another phase. This phenomenon is known as martensitic transformation. Such forward and reverse phase transformations form the base for the unique behavior of SMAs.

The Shape Memory Effect (SME) phenomenon is responsible for the macroscopic shape change of the alloy and its complete recovery during phase transformation.[2] Under zero load condition, when the temperature is at the martensitic start temperature (M_s), austenite begins to transform to twinned martensite and completes the transformation to martensite at the martensitic finish temperature (M_f); this is known as forward transformation. The transformation is complete at this stage, and the material is entirely in the twinned martensite phase. In an equivalent manner, the reverse transformation occurs during the heating process; it starts at the austenitic start temperature (A_s) and gets completed at the austenitic finish temperature (A_f). Under the applied stress condition, the low-temperature twinned martensitic phase is converted to detwinned martensite due to reorientation. The macroscopic shape change is a result of the detwinning process, retaining the deformed configuration when the stress is relieved. Subsequent heating of the SMA to a temperature above A_s results in a change of phase from detwinned martensite to austenite (reverse phase transformation), leading to complete shape recovery. When the temperature falls below M_s, twinned martensite formation occurs without any shape change observed during forward transformation.

1.2 Market access information

According to Fortune Business Insights, the global market size of actuators was $39.08 billion in 2020 and is estimated to reach $91.05 billion by 2028 with a CAGR of 12.06% during 2021-2028 period[3]. Furthermore, as per a report by Markets and Markets, the actuators market is expected to grow from $53.9 billion in 2021 to $86.6 billion by 2027 at a CAGR of 8.2%[4]. The actuator market is segmented on the type of actuator, basis of actuation, vertical, region, and application. The increased investments in oil and gas, power, chemicals, and the rising adoption of international safety standards and practices are expected to influence actuator market growth in the Asia-Pacific region. The actuator market is forecasted to rise at a healthy rate, attributed to an increase in automated systems in industrial applications. With increased focus towards automation of production operations for increased precision, enhanced safety, and higher efficiency is expected to fuel market growth. The rising demand for advanced actuator products is compelling OEMs to invest in R&D activities.

1.3 Comparison with existing SMA-based actuators

Table S1 shows that SMA-based actuators available in the open literature reported actuation force lesser than 30 N with the power consumption at par or more significant than reported in the submitted manuscript [5–7]. The parallel arrangement-based linear actuators reported the SMA wire length in the range of 350 mm, and hence its overall envelop packaging exceeds 350 mm [6,7]. The proof-of-concept bipennate-based SMA actuator utilizes 1000 mm of SMA wire for actuation; however, by virtue of the bipennate arrangement, the SMA wires are accommodated within the overall dimensions of 390 mm x 95 mm x 15 mm using an extension type bias spring. In another embodiment with a compression bias spring, a similar bipennate-based linear actuator can be confined within 280 mm length. As evident from Table S1, on average, the net actuation force produced by the bipennate-based SMA actuator is four times more than other SMA-based actuator designs with half the power consumption. The force produced in a single fiber causes macro-level muscle force generation in bipennate musculature. Thus, the current system also gives the functionality of adding even more unipennate SMA branches in the stipulated length. Hence, an even higher force can be generated in the same actuator dimension.

On the other hand, while the SMA-based actuators available in the literature reported a much higher force, as shown in Table S1, they had significantly higher power consumption than the SMA-based linear actuation system with a bipennate configuration. Upon comparison, Mosley et al. (1999)[8] had a parallel configuration of 48 SMA wire bundles, with approximately 15 times the SMA length and close to 11 times the power supplied, to yield a force approx. 4.5 times the force generated by the current bipennate based SMA actuator. Similarly, Shin et al. (2005)[9] required approximately 150 times the power supplied to yield a force closer to 2 times the force reported in the submitted manuscript, as shown in Table S1. The present bio-inspired shape memory alloy-based hierarchical actuator produces a maximum measured force of 105 N when actuated by a power supply of 7 V pulse voltage with a current of 1.6 A, and the corresponding power consumption is 11.2 W.

Research articles	SMA based actuator designs	Force output	Power consumption	Length of SMA wire
Chaurasiya et al. (Proposed prototype)	Bipennate based SMA actuator	100 N	11.4 W	1000 mm
Yuen et al.[5]	Embedded actuator with robotic fabric	9.6 N	12 W (max)	1286 mm
Reynearts et al.[6]	Shape memory alloy actuator	21-27 N	30 W	360 mm
Lee et al.[7]	Tendon-based soft robotics actuator	25-30 N	–	350 mm
Mosley et al.[8]	SMA bundle actuator	445 N	126 W	48 SMA wire bundles
Shin et al.[9]	Hydraulic linear actuator with thin film SMA	198 N	20 V 80 A	–

Table S1. Comparative analysis of the proposed SMA-based linear actuation system with a bipennate configuration vis-a-vis with existing SMA-based actuators reported in literature. '–' denotes that the data was not available in the reported article.

2 Supplementary Results and Discussion

2.1 Case study for variable input voltage pulse

The simulation is carried out for variable voltage input condition with the pulse varying every 10 sec for a total of 60 sec as shown in Figure S1a. The actuator force was studied along with the variation of stress with temperature and variation of martensite volume fraction with temperature in the Figure S2b, S2d, and S2f respectively.

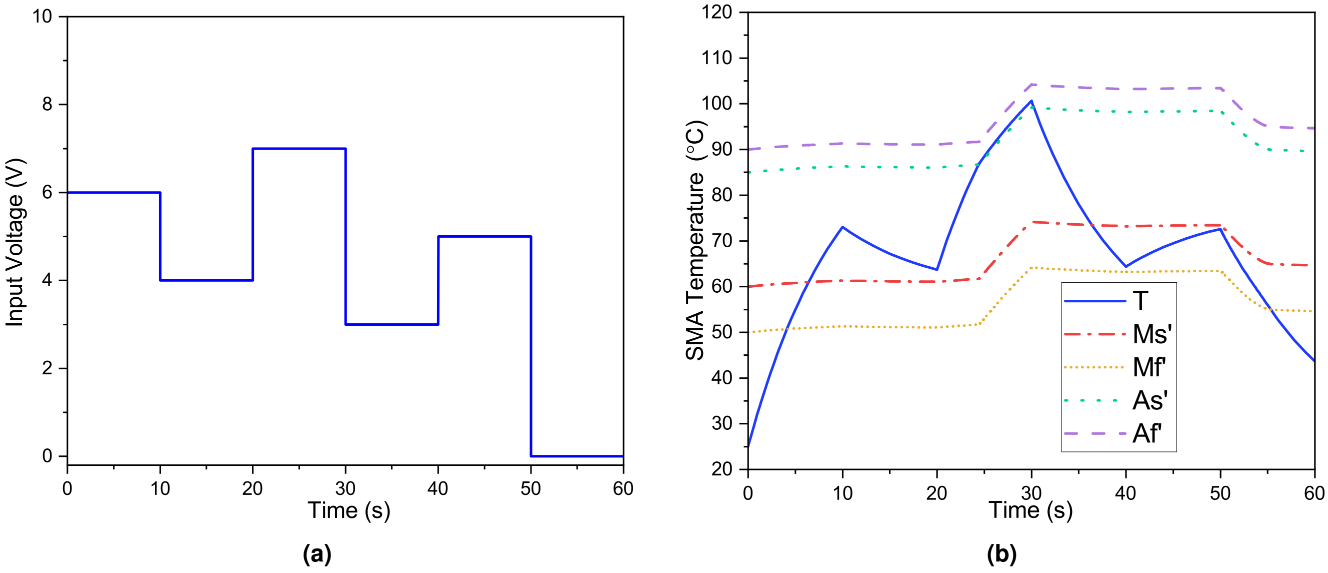

Figure S1. (a) For this simulation, a variable input voltage was considered. For the first 10 sec, 6 V was applied, then 4 V, 7 V, 3 V, 5 V, and finally 0 V for 10 sec each, (b) displays the simulation output of the temperature distribution as well as the stress-induced transition temperature of the SMA-based bipennate actuator. The temperature of the wire is directly proportional to the voltage applied across the wire. When the temperature of the wire crosses the materials austenite transition temperature in the heating phase, the modified austenite transition temperature starts to rise, and likewise happens with martensite transition temperatures when the temperature of the wire crosses the materials martensite transition temperature in the cooling phase.

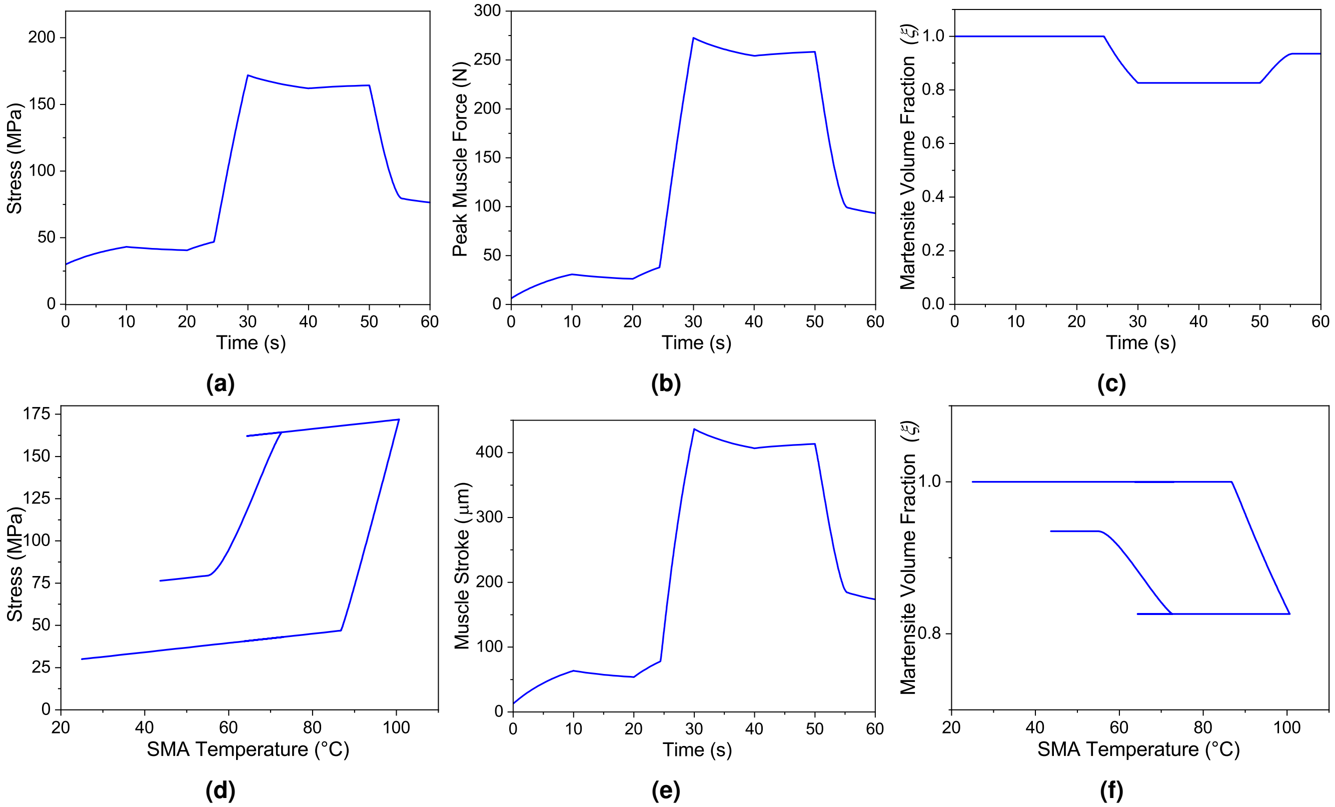

Figure S2. Under the input condition of as illustrated in Figure S1a, analytical findings for the distribution of various parameters are displayed. While (a), (b), (c) and (e) depicts a temporal distribution, on the other hand (d) and (f) illustrates distribution over temperature. For the concerned input condition, the maximum stress observed was 171 MPa (which was less than 345 MPa, the yield strength of the wire), with a force of 272 N and a maximum displacement of 436 μm, and the minimum martensite volume fraction was 0.82. On the other hand, the variation of stress and variation of martensite volume fraction with temperature resembles the hysteresis property.

2.2 Sensitivity analysis

In order to understand the influence of the physical parameters on the force output of the actuator, the sensitivity analysis study of the multi-field coupled mathematical model, as shown in Figure S6, has been carried out on the selected physical parameters to rank the parameters in the order of their influence. The affect of model parameters on the output force was observed using 2500 unique set of values which were generated using different probability distribution as shown in the Figure S3. The peak muscle force was selected as the design requirement for the study and the parametric influence of each set of variables on the force generation is shown in the scatter plot, figure Figure S4. A tornado plot is obtained from the sensitivity analysis in terms of correlation coefficients for each parameter (refer Figure 6a of main article file). V_{in} (input voltage), K_x (spring constant), α (angle of pennation), h_T (convective heat transfer coefficient), l_0 (initial length of SMA), and n (number of unipennate branches) were the variables used for sensitivity analysis. Except for n, which was chosen at random from a multinomial probability distribution with equal probability for all even numbers from 4 to 24, all other parameters were chosen at random from a uniform probability distribution (the range of parameters has been illustrated in Figure S3).

The simulation is performed for voltage pulse of variable input voltage condition as shown in Figure S1a. The voltage pulse consists of series of step up and down in between spanning for the total time duration of 60 sec. Figure S1b depicts the temperature response of SMA wires over time in response to a varied input voltage pulse. As shown in Figure S1b, the wire temperature rises and remains below the critical point of stress-modified austenite phase start temperature (A_s') during the first 10 sec. As a result, the martensite volume fraction remains unchanged during this time because it does not reach the transformation zone. The reverse transformation occurs when the SMA temperature crosses the critical point A_s' at $t = 23.3$ sec. The joule heating provided to the SMA wires from $t = 20$ to 30 sec is sufficient enough to allow the SMA to reach in the transformation zone. Subsequently, stress is generated in the SMA wire as a consequence of the reduction in the the martensite volume fraction as well as stroke output in the actuator is produced as shown in Figure S2a, S2c and S2e respectively. The

SMA wire temperature keeps on increasing as the heat supply is provided followed by convective cooling during zero voltage condition. During heating, when the SMA wire temperature (T) crosses the stress-modified austenite phase start temperature (A'_s), the reverse transformation from martensite to austenite phase starts to occur. At this stage, the SMA wire contracts and the muscle force is generated by the actuator.

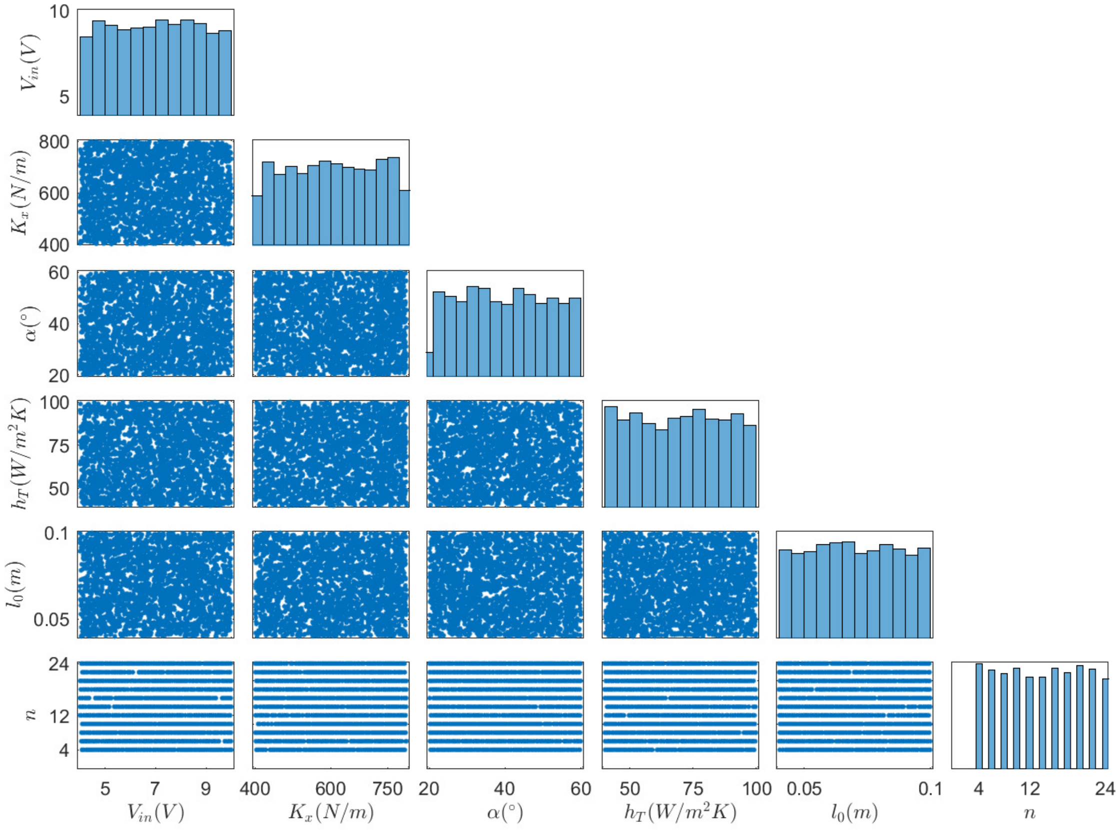

Figure S3. Multiple probabilistic distributions were used to generate 2500 unique set of the mentioned model parameters. While n was generated using multinomial distribution with each unique value having the same probability of occurrence, rest of the model parameters were generated using uniform distribution. K_x ranges from $400-800$ N/m, while n ranges from $4-24$. Voltage (V_{in}) has been altered from $4-10$ V, initial wire length (l_0) has been varied from $40-100$ mm, convective heat transfer co-efficient (h_T) has been varied from $50-100$ W/m$^2-$K and pennation angle (α) has been varied from $20-60^\circ$.

Figure S2a and S2b show the stress induced in the SMA wire and the force generated by the actuator with respect to time. During reverse transformation (heating), when the SMA wire temperature, $T < A'_s$, the rate of change of martensite volume fraction ($\dot{\xi}$) will be zero as given in equation (S5). Therefore, the rate of change of stress ($\dot{\sigma}$) will depend on the strain rate ($\dot{\varepsilon}$) and the temperature gradient ($\dot{T}$) only as given by equation (2) (refer Constitutive equation section in the main article file). However, as the SMA wire temperature increases and crosses (A'_s), the austenite phase starts to form and the ($\dot{\xi}$) takes a value as given by equations (S1), (S3) and (S4). Hence, the stress change rate ($\dot{\sigma}$) is collectively governed by $\dot{\varepsilon}, \dot{T}$ and $\dot{\xi}$ as given in equation (2) (refer Constitutive equation section in the main article file). This explains the change in the gradient observed in the time-dependent stress and force plots during the heating cycle as shown in Figure S2a and S2b. The same explanation holds good for the forward transformation (cooling) from austenite to martensite phase with the relation between the SMA wire temperature (T) and the stress-modified martensite phase finish temperature (M'_f) as well. Figure S2d and S2f shows the variation of the stress induced in SMA wire (σ) and the martensite volume fraction (ξ) with respect to change in the SMA wire temperature (T) for two actuation cycles.

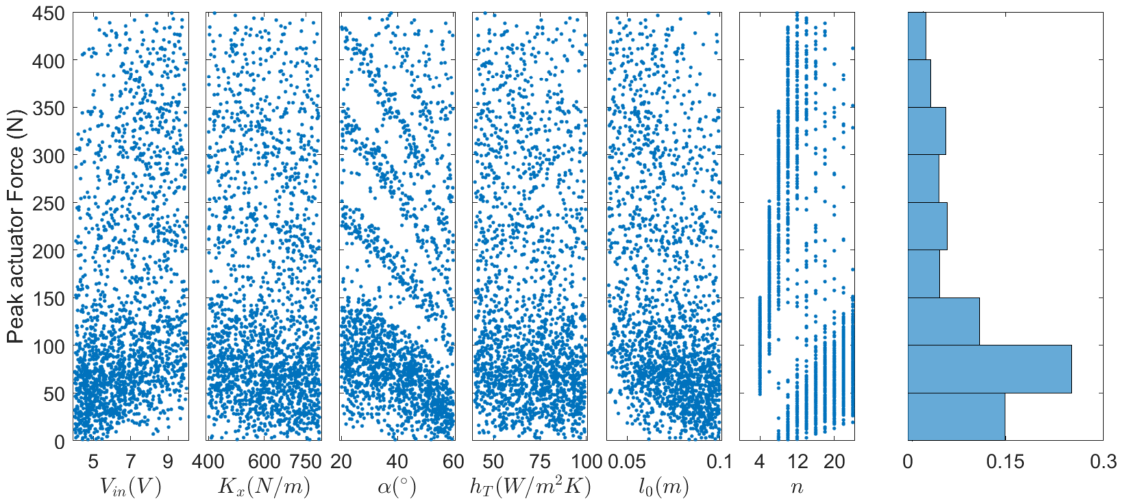

Figure S4. The figure depicts the distribution of the peak muscle force attributed to different combinations of model parameters. As evident from the figure, the majority of the 2500 distinct combinations picked generate forces less than 150 N, with forces larger than 150 N having a smaller but equal representation. Meanwhile, each points in the parameter space denotes one unique value of that parameter.

Figure S3 shows the parameter sample space distributions for the parameters chosen for the sensitivity analysis study. For a set of six model parameters, 2500 unique set of values have been generated to study their influence on the maximum force output as shown in the Figure S4. Figure 6a (refer to main article file) shows the tornado plot of the various correlation coefficients for each parameter against the design requirement of maximum output force. It can be observed from the Figure 6a (refer to main article file) that the parameters, voltage (V_{in}) is directly correlated with the maximum output force, while the initial length of SMA wire (l_0), number of unipennate wires (n), convective heat transfer coefficient (h_T), pennation angle (α), bias spring constant (K_x) are inversely correlated with the output force. In case of direct correlation, the higher value of the correlation coefficient for the voltage (V_{in}) signifies that this parameter influences the force output the most while initial length of SMA wire (l_0) is most inversely correlated. The analysis helps in identifying and customizing the influential parameters through which the output force, stroke and efficiency of the actuator system can be tailored as per the requirement and application.

2.3 Effect of input voltage and forced convection on actuation frequency

This study elucidates the effect of external stimuli (V_{in} and h_T) on the actuation frequency of the bipennate-based SMA actuator and the necessary parameter tuning required to achieve the actuation frequency as per the requirement and application. The simulation result reported in this paper was performed with a supply voltage (V_{in}) of 7 V and a forced convection condition (h_T=70 W/m^2-K). The input voltage was supplied for 10 sec during the heating cycle, followed by a 15-second cooling period. Thus, it resulted in an actuation cycle of 25 sec or a frequency of 0.04 Hz. By performing simulation, the improvement in the actuation frequency has been observed by reducing the heating cycle period by increasing the input voltage from 7V to 15V and simultaneously enhancing the forced convection, from 300 to 500 W/m^2-K, to reduce the cooling cycle period. Figure S5d shows the input conditions of the different sets for the simulation and corresponding output in terms of the frequency, heating time, and cooling time period. Furthermore, Figure S5a and S5b show the variation in actuation force and SMA wire temperature for different sets of input voltage and convective heat transfer coefficients. Figure S5c illustrates a 303% increase in the actuation frequency upon increasing the input voltage to 15 V and forced convection to 500 W/m^2-K simultaneously.

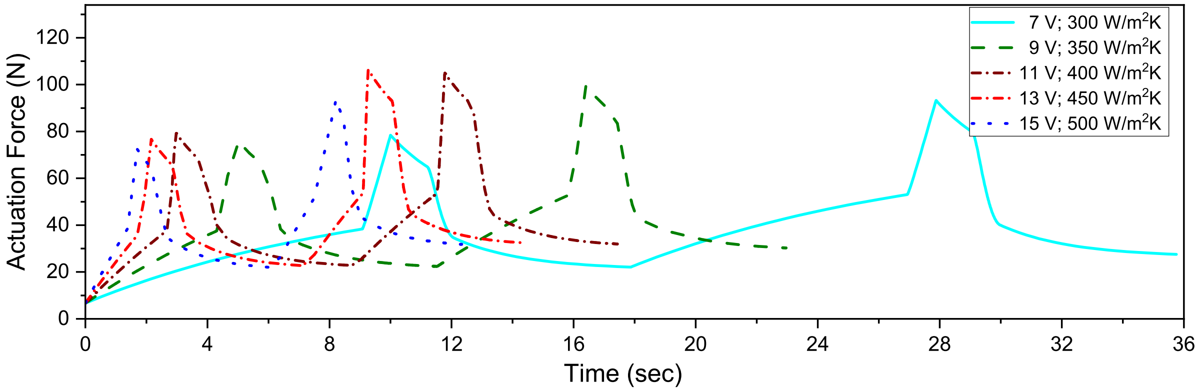

(a)

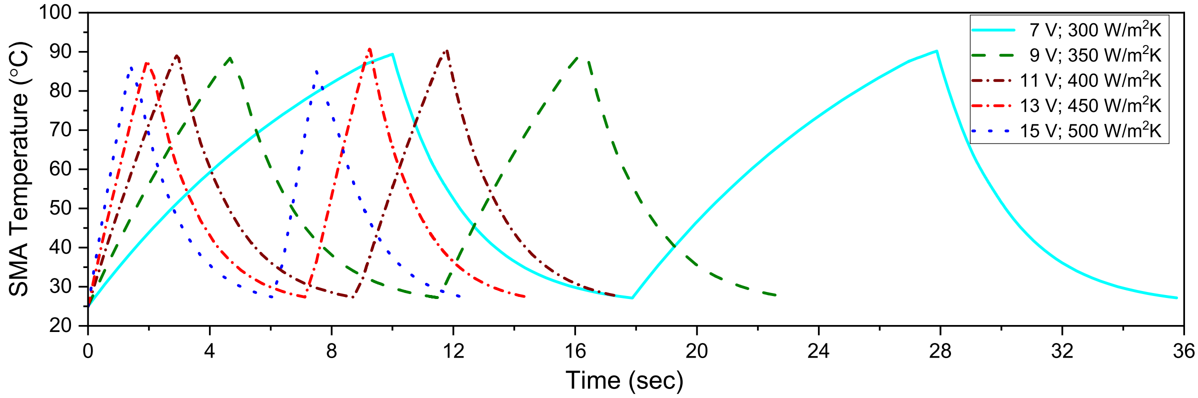

(b)

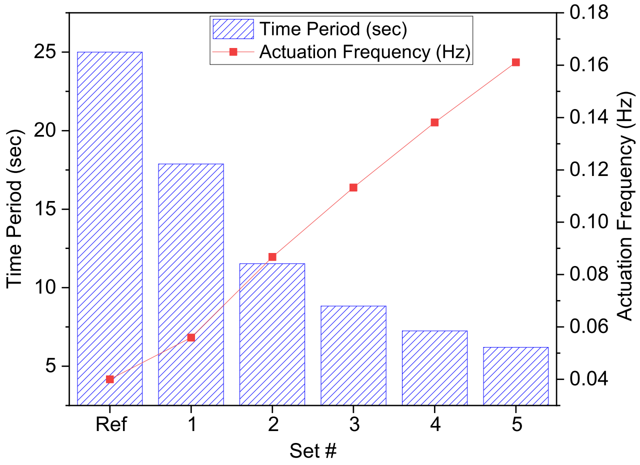

(c)

Set #	V_{in} (V)	h_T $(Wm^{-2}K^{-1})$	t_H (sec)	t_C (sec)	f (Hz)
Ref	7	70	10.0	15.0	0.04
1	7	300	10.0	7.8	0.05
2	9	350	4.8	6.7	0.08
3	11	400	2.9	5.8	0.11
4	13	450	2.0	5.2	0.13
5	15	500	1.4	4.7	0.16

(d)

Figure S5. (a), (b) The effect of input voltage (V_{in}) and forced convection (h_T) on the actuation force and SMA wire temperature is illustrated. The input voltage was increased during the heating cycle to reduce the heating time, wherein wires were heated till 89°C approximately in the first cycle for each set. On the other hand, forced convection was increased during the cooling cycle to reduce the cooling period, wherein the wires were allowed to cool to approximately 27°C in the first cooling cycle for each set. (c) depicts the effect on actuation frequency and the actuation time period. It is imperative to note that "Ref" refers to the parameter set in the main article and is considered to be the benchmark set. It has been observed that the time period has an inverse correlation, whereas the actuation frequency is positively correlated with the input voltage and convective heat transfer coefficient. (d) Table illustrates the effect of the V_{in} and h_T on heating time (t_H), cooling time (t_C) and actuation frequency. A 303% increase in actuation frequency was observed in Set 5 with respect to the benchmark set.

3 Supplementary Methods

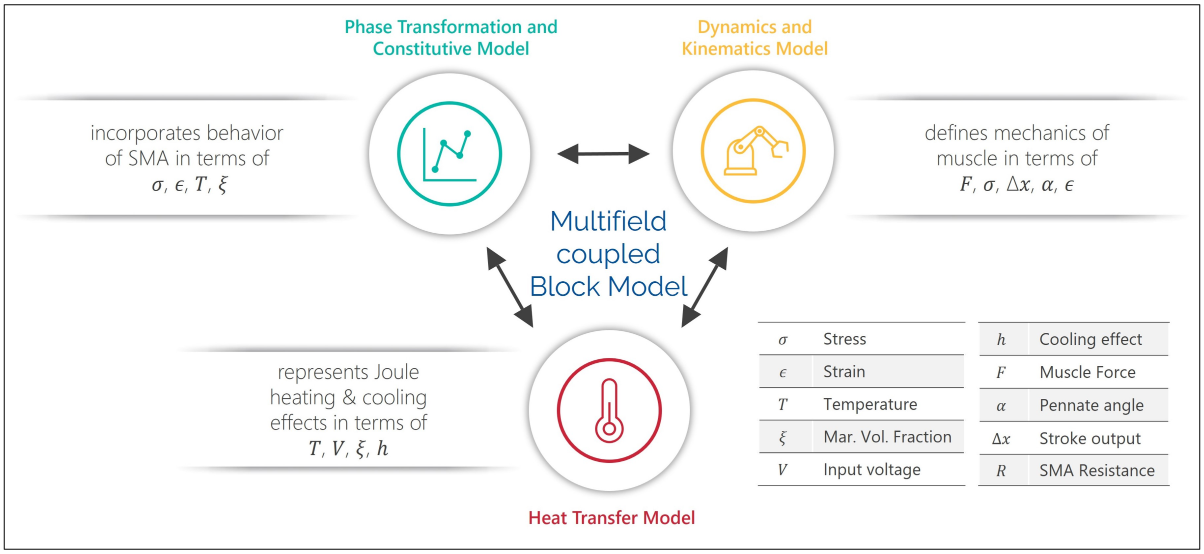

Figure S6. Schematic depicting multi-field coupled block diagram of SMA based bipennate actuation system. The output of one block acts as the input to the other. The heat transfer model is in itself a coupled model where the heat transferred into the wire is done by the Joule heating phenomenon. The phase transformation and constitutive model incorporates the physics of SMA while the dynamics and kinematics model takes care of the kinematics and mechanics of the actuator.

3.1 Phase transformation equations

The time dependent martensite volume fraction can be expressed as a function of stress rate and temperature gradient in the following manner:

$$\dot{\xi} = \eta_\sigma \dot{\sigma} + \eta_T \dot{T} \tag{S1}$$

The conditions to be satisfied for the enhanced phenomenological model[10] during phase transformation are as follows:

3.1.1 Reverse transformation (Martensite to Austenite)

The governing equation of reverse transformation represented by the phase change from martensite to austenite during heating condition is given by:

$$\xi = \frac{\xi_M}{2}\left[cos\left[a_A\left(T - A_s\right) + b_A\sigma\right] + 1\right] \tag{S2}$$

$$\text{if } \dot{T} - \frac{\dot{\sigma}}{C_A} > 0 \text{ and } A'_s = \left(A_s + \frac{\sigma}{C_A}\right) \leq T \leq \left(A_f + \frac{\sigma}{C_A}\right) = A'_f$$

$$\eta_\sigma = \frac{\xi_M}{2}\left(\frac{a_A}{C_A}\right)\sin\left[a_A\left(T - A_s - \frac{\sigma}{C_A}\right)\right] \tag{S3}$$

$$\eta_T = -\frac{\xi_M}{2}\left(a_A\right)\sin\left[a_A\left(T - A_s - \frac{\sigma}{C_A}\right)\right] \tag{S4}$$

else,

$$\dot{\xi} = 0 \tag{S5}$$

where, A'_s and A'_f are stress-modified austenite phase start and finish temperatures, respectively.

3.1.2 Forward transformation (Austenite to Martensite)

The governing equation of forward transformation represented by the phase change from austenite to martensite during cooling condition is given by:

$$\xi = \frac{1-\xi_A}{2} cos\left[a_M\left(T-M_f\right)+b_M\sigma\right]+\frac{1+\xi_A}{2} \tag{S6}$$

$$\text{if } \dot{T}-\frac{\dot{\sigma}}{C_M}<0 \text{ and } M_f' = \left(M_f+\frac{\sigma}{C_M}\right) \leq T \leq \left(M_s+\frac{\sigma}{C_M}\right) = M_s'$$

$$\eta_\sigma = \left(\frac{1-\xi_A}{2}\right)\left(\frac{a_M}{C_M}\right)\sin\left[a_M\left(T-M_f-\frac{\sigma}{C_M}\right)\right] \tag{S7}$$

$$\eta_T = -\left(\frac{1-\xi_A}{2}\right)(a_M)\sin\left[a_M\left(T-M_f-\frac{\sigma}{C_M}\right)\right] \tag{S8}$$

else,

$$\dot{\xi} = 0 \tag{S9}$$

where, M_s' and M_f' are stress-modified martensite phase start and finish temperatures, respectively.

3.2 Bipennate muscle stiffness equation – 1st hierarchical level actuator

The total force generated by the SMA based bipennate muscle arrangement is given as follows:

$$F_m = nF_f\cos\alpha - K_x(\Delta x + x_0) \tag{S10}$$

where, n is the number of unipennate branches, F_m is the muscle force generated by the actuator, F_f is the fiber force in the SMA wire, K_x is the stiffness of the bias spring, Δx is the stroke of the actuator, α is the angle of pennation and x_0 is the initial displacement of the bias spring to maintain the SMA wires in the pre-tension arrangement. Considering the stroke (Δx) of the muscle to be small as compared to x_0, the mechanics equation reduces to the following form,

$$F_m = nF_f\cos\alpha - K_x x_0 \tag{S11}$$

The stiffness of the pennate muscle (k_m) can be defined as the derivative of the total muscle force generated with respect to the muscle position[11]. Upon differentiating equation (S11) with respect to muscle position, the pennate muscle stiffness (k_m) is given by:

$$k_m = \frac{dF_m}{dx} = n\left[\left(\frac{x}{l}\right)\frac{dF_f}{dx}+\frac{F_f}{l}+F_f\left(-\frac{x}{l^2}\right)\frac{dl}{dx}\right] \tag{S12}$$

where, $\cos\alpha = x/l$ as shown in Figure 9d (refer main article file).

Upon applying the chain rule of differentiation, the equation (S12) is modified as follows:

$$k_m = n\left[\left(\frac{x}{l}\right)\frac{dF_f}{d\varepsilon}\cdot\frac{d\varepsilon}{dl}\cdot\frac{dl}{dx}+\frac{F_f}{l}-F_f\left(\frac{x}{l^2}\right)\frac{dl}{dx}\right] \tag{S13}$$

where,

$$\varepsilon = \frac{l_0-l}{l_0} \Rightarrow \frac{d\varepsilon}{dl} = -\frac{1}{l_0} \tag{S14}$$

$$\frac{dl}{dx} = \frac{x}{\sqrt{x^2+t^2}} = \frac{x}{l} \tag{S15}$$

Substituting the values from equations (S14) and (S15) into equation (S13) and simplifying it further, the pennate muscle stiffness (k_m) is modified as follows:

$$k_m = n\left[-\frac{1}{l_0}\left(\frac{x}{l}\right)^2\frac{dF_f}{d\varepsilon}+\frac{F_f}{l}-\frac{F_f}{l}\left(\frac{x}{l}\right)^2\right] \tag{S16}$$

$$\Rightarrow k_m = n\left[-\left(\frac{1}{l_0}\right)\frac{dF_f}{d\varepsilon}\cos^2\alpha + \frac{F_f}{l}\sin^2\alpha\right] \tag{S17}$$

$$\Rightarrow k_m = \frac{n}{l_0}\left(-\frac{dF_f}{d\varepsilon}\cos^2\alpha + \frac{F_f}{1-\varepsilon}\sin^2\alpha\right) \tag{S18}$$

$$\Rightarrow k_m = \frac{n}{l_0}\left(-\frac{d\sigma}{d\varepsilon}A_{cross}\cos^2\alpha + \frac{\sigma}{1-\varepsilon}A_{cross}\sin^2\alpha\right) \tag{S19}$$

where, $F_f = \sigma A_{cross}$, $E = d\sigma/d\varepsilon$, σ is the stress induced in the SMA wire, and A_{cross} is the cross-sectional area of the SMA wire,

$$\therefore k_m = \frac{n}{l_0}A_{cross}\left(-E\cos^2\alpha + \frac{\sigma}{1-\varepsilon}\sin^2\alpha\right) \tag{S20}$$

Combining equations (S11), (S12) and (S20), it can be written as:

$$k_m\Delta x = n\sigma A_{cross}\cos\alpha - K_x x_0 \tag{S21}$$

$$\Rightarrow \left[\frac{n}{l_0}A_{cross}\left(-E\cos^2\alpha + \frac{\sigma}{1-\varepsilon}\sin^2\alpha\right)\right]\Delta x = n\sigma A_{cross}\cos\alpha - K_x x_0 \tag{S22}$$

The total displacement or stroke (Δx) of the 1st level hierarchical actuator as a function of stress induced (σ) and strain developed (ε) in the SMA wire is given by:

$$\Delta x = \frac{n\sigma A_{cross}\cos\alpha - K_x x_0}{\frac{n}{l_0}A_{cross}\left[-E\cos^2\alpha + \frac{\sigma}{1-\varepsilon}\sin^2\alpha\right]} \tag{S23}$$

3.3 Bipennate muscle stiffness equation – 2nd hierarchical level actuator

The total force generated by the 2nd level SMA based bipennate muscle arrangement is given as follows:

$$F_m = n_1 n_2 F_f \cos\alpha_1 \cos\alpha_2 - K_x(\Delta x_2 + x_0) \tag{S24}$$

where, n_1 denotes the number of unipennate SMA wire branches, and n_2 denotes the number of secondary arms of the actuator.

Considering the stroke (Δx_2) of the muscle to be small as compared to x_0, the mechanics equation reduces to the following form,

$$F_m = n_1 n_2 F_f \cos\alpha_1 \cos\alpha_2 - K_x x_0 \tag{S25}$$

The stiffness of the pennate muscle (k_m) can be defined as the derivative of the total muscle force generated with respect to the position. Upon differentiating equation (S25) with respect to muscle position, the pennate muscle stiffness (k_m) is given by:

$$k_m = \frac{dF_m}{dx_2} = \frac{d}{dx_2}\left[n_1 n_2 F_f \cos\alpha_1 \cos\alpha_2 - K_x x_0\right] \tag{S26}$$

Applying chain rule to equation (S26), the modified form is given as follows:

$$\frac{dF_m}{dx_2} = n_1 n_2\left[\cos\alpha_1\cos\alpha_2\frac{dF_f}{dx_2} + F_f\cos\alpha_1\frac{d}{dx_2}(\cos\alpha_2) + F_f\cos\alpha_2\frac{d}{dx_2}(\cos\alpha_1)\right] \tag{S27}$$

As per the illustration in Figure S7 and using the similar approach as of equation (S15), the following differential and trigonometrical relations can be obtained:

$$\frac{dl_1}{dx_1} = \frac{x_1}{l_1} = \cos\alpha_1 \tag{S28}$$

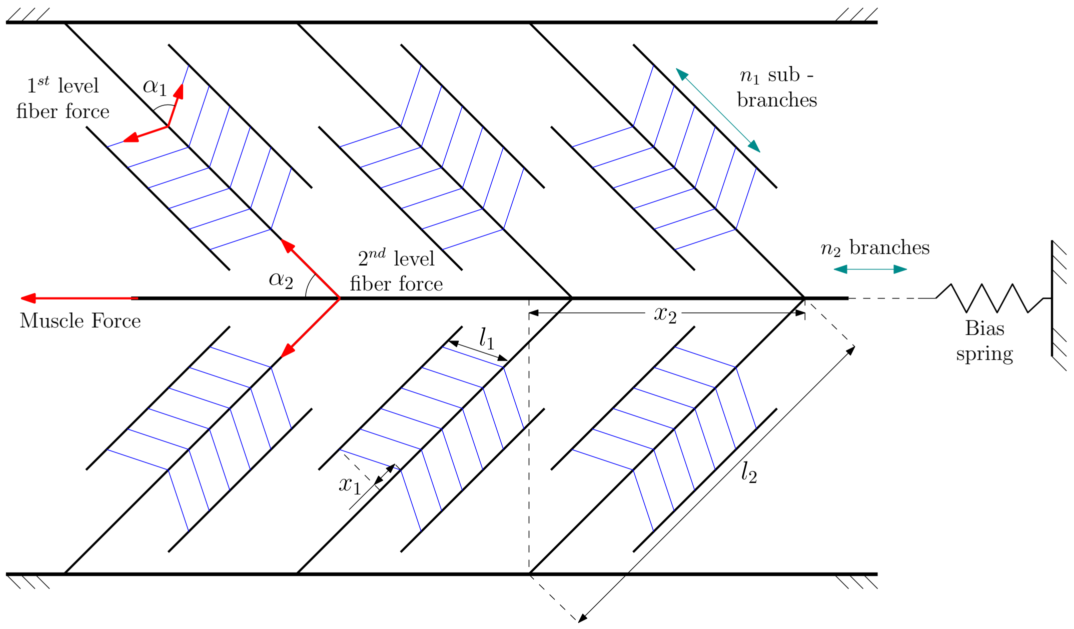

Figure S7. The 2nd level actuator is shown schematically. The angle subtended with the arm is denoted by α_1 and α_2, where α_1 is the angle SMA wire has with the arm attached (secondary arm) and α_2 is the angle the secondary arm has with the primary arm. Similarly, n_1 represents the number of SMA wires in each arm, whereas n_2 represents the number of secondary arms connected to the primary arm. On the other hand, the projection of the SMA wire on the secondary arm is x_1, while the projection of the secondary arm on the primary arm is x_2.

$$\frac{dl_2}{dx_2} = \frac{x_2}{l_2} = \cos\alpha_2 \tag{S29}$$

Substituting the values from equations (S28) and (S29), the equation (S27) can be modified as:

$$\frac{dF_m}{dx_2} = n_1 n_2 \left[\cos\alpha_1 \cos\alpha_2 \frac{dF_f}{dx_2} + F_f \cos\alpha_1 \frac{d}{dx_2}\left(\frac{x_2}{l_2}\right) + F_f \cos\alpha_2 \frac{d}{dx_2}\left(\frac{x_1}{l_1}\right)\right] \tag{S30}$$

$$\Rightarrow \frac{dF_m}{dx_2} = n_1 n_2 \left[\cos\alpha_1 \cos\alpha_2 \frac{dF_f}{d\varepsilon} \cdot \frac{d\varepsilon}{dl_1} \cdot \frac{dl_1}{dx_2} + F_f \cos\alpha_1 \left(\frac{1}{l_2} - \frac{x_2}{l_2^2} \cdot \frac{dl_2}{dx_2}\right) + F_f \cos\alpha_2 \left(\frac{1}{l_1} \cdot \frac{dx_1}{dx_2} - \frac{x_1}{l_1^2} \cdot \frac{dl_1}{dx_2}\right)\right] \tag{S31}$$

$$\Rightarrow \frac{dF_m}{dx_2} = n_1 n_2 \left[\cos\alpha_1 \cos\alpha_2 \frac{dF_f}{d\varepsilon} \cdot \frac{d\varepsilon}{dl_1} \cdot \frac{dl_1}{dx_1} \cdot \frac{dx_1}{dx_2} + \frac{F_f}{l_2} \cos\alpha_1 \left(1 - \frac{x_2^2}{l_2^2}\right) + \frac{F_f}{l_1} \cos\alpha_2 \left(1 - \frac{x_1}{l_1} \cdot \frac{dl_1}{dx_1}\right) \frac{dx_1}{dx_2}\right] \tag{S32}$$

$$\Rightarrow \frac{dF_m}{dx_2} = n_1 n_2 \left[\frac{x_1}{l_1} \cos\alpha_1 \cos\alpha_2 \frac{dF_f}{d\varepsilon} \cdot \frac{d\varepsilon}{dl_1} \cdot \frac{dx_1}{dx_2} + \frac{F_f}{l_2} \cos\alpha_1 \left(1 - \frac{x_2^2}{l_2^2}\right) + \frac{F_f}{l_1} \cos\alpha_2 \left(1 - \frac{x_1^2}{l_1^2}\right) \frac{dx_1}{dx_2}\right] \tag{S33}$$

As shown in Figure S7, the geometrical constraint of the displacements of the primary and secondary arm is given by:

$$\Delta x_2 = \Delta x_1 \cos\alpha_2 \tag{S34}$$

Upon applying the first principle of derivative on elemental displacement, the following relation can be obtained:

$$\frac{dx_1}{dx_2} = \lim_{\substack{\Delta x_1 \to 0 \\ \Delta x_2 \to 0}} \left(\frac{\Delta x_1}{\Delta x_2}\right) = \sec\alpha_2 \tag{S35}$$

$$\varepsilon = \frac{l_{10} - l_1}{l_{10}} \Rightarrow \frac{d\varepsilon}{dl_1} = -\frac{1}{l_{10}} \tag{S36}$$

Substituting the values from equations (S35) and (S36), the equation (S33) can be modified as:

$$\frac{dF_m}{dx_2} = n_1 n_2 \left[\cos^2\alpha_1 \cos\alpha_2 \frac{dF_f}{d\varepsilon}\left(-\frac{1}{l_{10}}\right)\sec\alpha_2 + \frac{F_f}{l_2}\cos\alpha_1\left(1-\cos^2\alpha_2\right) + \frac{F_f}{l_1}\cos\alpha_2\left(1-\cos^2\alpha_1\right)\sec\alpha_2\right] \tag{S37}$$

$$\Rightarrow \frac{dF_m}{dx_2} = n_1 n_2 \left[-\frac{1}{l_{10}}\cos^2\alpha_1 \frac{dF_f}{d\varepsilon} + \frac{F_f}{l_2}\cos\alpha_1 \sin^2\alpha_2 + \frac{F_f}{l_1}\sin^2\alpha_1\right] \tag{S38}$$

$$\Rightarrow \frac{dF_m}{dx_2} = n_1 n_2 \left[-\frac{1}{l_{10}}A_{cross}\cos^2\alpha_1 \frac{d\sigma}{d\varepsilon} + \frac{\sigma}{l_2}A_{cross}\cos\alpha_1 \sin^2\alpha_2 + \frac{\sigma}{l_{10}(1-\varepsilon)}A_{cross}\sin^2\alpha_1\right] \tag{S39}$$

where, $F_f = \sigma A_{cross}$, $E = d\sigma/d\varepsilon$, σ is the stress induced in the SMA wire, $l_1 = l_{10}(1-\varepsilon)$ and A_{cross} is the cross-sectional area of the SMA wire,

$$\therefore \frac{dF_m}{dx_2} = n_1 n_2 A_{cross}\left[-\frac{E}{l_{10}}\cos^2\alpha_1 + \frac{\sigma}{l_2}\cos\alpha_1 \sin^2\alpha_2 + \frac{\sigma}{l_{10}(1-\varepsilon)}\sin^2\alpha_1\right] \tag{S40}$$

Combining equations (S25), (S26) and (S40) it can be written as:

$$k_m \Delta x_2 = n_1 n_2 \sigma A_{cross}\cos\alpha_1 \cos\alpha_2 - K_x x_0 \tag{S41}$$

$$\Rightarrow n_1 n_2 A_{cross}\left[-\frac{E}{l_{10}}\cos^2\alpha_1 + \frac{\sigma}{l_2}\cos\alpha_1 \sin^2\alpha_2 + \frac{\sigma}{l_{10}(1-\varepsilon)}\sin^2\alpha_1\right]\Delta x_2 = n_1 n_2 \sigma A_{cross}\cos\alpha_1\cos\alpha_2 - K_x x_0 \tag{S42}$$

The total displacement or stroke (Δx_2) of the 2nd level hierarchical actuator as a function of stress induced (σ) and strain developed (ε) in the SMA wire is given by:

$$\Delta x_2 = \frac{n_1 n_2 \sigma A_{cross}\cos\alpha_1\cos\alpha_2 - K_x x_0}{n_1 n_2 A_{cross}\left[-\frac{E}{l_{10}}\cos^2\alpha_1 + \frac{\sigma}{l_2}\cos\alpha_1\sin^2\alpha_2 + \frac{\sigma}{l_{10}(1-\varepsilon)}\sin^2\alpha_1\right]} \tag{S43}$$

3.4 Bipennate muscle stiffness equation – 3rd hierarchical level actuator

The total force generated by the 3rd level SMA based bipennate muscle arrangement is given as follows:

$$F_m = n_1 n_2 n_3 F_f \cos\alpha_1 \cos\alpha_2 \cos\alpha_3 - K_x\left(\Delta x_3 + x_0\right) \tag{S44}$$

where, n_1 represents the number of SMA wires in each 1st level actuator-like structure, n_2 represents the number of 1st level actuators coupled to each secondary arm, and n_3 represents the total number of secondary arms. On the other hand, α_i represents the angle subtended to the arms, where $i = (N = 3)$ (in case of 3rd level actuator) denotes angle subtended to the primary arm and $i = N - 1$ denotes the angle subtended to the secondary arm and so on.

Considering the stroke (Δx_3) of the muscle to be small as compared to x_0, the mechanics equation reduces to the following form,

$$F_m = n_1 n_2 n_3 F_f \cos\alpha_1 \cos\alpha_2 \cos\alpha_3 - K_x x_0 \tag{S45}$$

The stiffness of the pennate muscle (k_m) can be defined as the derivative of the total muscle force generated with respect to the position. Upon differentiating equation (S45) with respect to muscle position, the pennate muscle stiffness (k_m) is given by:

$$k_m = \frac{dF_m}{dx_3} = \frac{d}{dx_3}\left[n_1 n_2 n_3 F_f \cos\alpha_1 \cos\alpha_2 \cos\alpha_3 - K_x x_0\right] \tag{S46}$$

Applying chain rule to equation (S46), the modified form is given as follows:

$$\frac{dF_m}{dx_3} = n_1 n_2 n_3 \Bigg[\cos\alpha_1 \cos\alpha_2 \cos\alpha_3 \frac{dF_f}{dx_3} + F_f \cos\alpha_1 \cos\alpha_2 \frac{d}{dx_3}(\cos\alpha_3) + F_f \cos\alpha_3 \cos\alpha_1 \frac{d}{dx_3}(\cos\alpha_2) + F_f \cos\alpha_2 \cos\alpha_3 \frac{d}{dx_3}(\cos\alpha_1)\Bigg] \tag{S47}$$

Extending the analogy similar to 2nd level actuator, the following differential and trigonometrical relations can be obtained for three-stage ($N = 3$) hierarchical actuator:

$$\frac{dl_1}{dx_1} = \frac{x_1}{l_1} = \cos\alpha_1 \tag{S48}$$

$$\frac{dl_2}{dx_2} = \frac{x_2}{l_2} = \cos\alpha_2 \tag{S49}$$

$$\frac{dl_3}{dx_3} = \frac{x_3}{l_3} = \cos\alpha_3 \tag{S50}$$

Substituting the values from equations (S48), (S49) and (S50), the equation (S47) can be modified as:

$$\frac{dF_m}{dx_3} = n_1 n_2 n_3 \Bigg[\cos\alpha_1 \cos\alpha_2 \cos\alpha_3 \frac{dF_f}{dx_3} + F_f \cos\alpha_1 \cos\alpha_2 \frac{d}{dx_3}\left(\frac{x_3}{l_3}\right) + F_f \cos\alpha_3 \cos\alpha_1 \frac{d}{dx_3}\left(\frac{x_2}{l_2}\right) + F_f \cos\alpha_2 \cos\alpha_3 \frac{d}{dx_3}\left(\frac{x_1}{l_1}\right)\Bigg] \tag{S51}$$

$$\Rightarrow \frac{dF_m}{dx_3} = n_1 n_2 n_3 \Bigg[\cos\alpha_1 \cos\alpha_2 \cos\alpha_3 \frac{dF_f}{d\varepsilon} \cdot \frac{d\varepsilon}{dl_1} \cdot \frac{dl_1}{dx_1} \cdot \frac{dx_1}{dx_3} + F_f \cos\alpha_1 \cos\alpha_2 \left(\frac{1}{l_3} - \frac{x_3}{l_3^2} \cdot \frac{dl_3}{dx_3}\right) + F_f \cos\alpha_3 \cos\alpha_1 \left(\frac{1}{l_2} \cdot \frac{dx_2}{dx_3} - \frac{x_2}{l_2^2} \cdot \frac{dl_2}{dx_3}\right) + F_f \cos\alpha_2 \cos\alpha_3 \left(\frac{1}{l_1} \cdot \frac{dx_1}{dx_3} - \frac{x_1}{l_1^2} \cdot \frac{dl_1}{dx_3}\right)\Bigg] \tag{S52}$$

$$\Rightarrow \frac{dF_m}{dx_3} = n_1 n_2 n_3 \Bigg[\cos\alpha_1 \cos\alpha_2 \cos\alpha_3 \frac{dF_f}{d\varepsilon} \cdot \frac{d\varepsilon}{dl_1} \cdot \frac{dl_1}{dx_1} \cdot \frac{dx_1}{dx_2} \cdot \frac{dx_2}{dx_3} + \frac{F_f}{l_3} \cos\alpha_1 \cos\alpha_2 \left(1 - \frac{x_3^2}{l_3^2}\right) + \frac{F_f}{l_2} \cos\alpha_3 \cos\alpha_1 \left(1 - \frac{x_2}{l_2} \cdot \frac{dl_2}{dx_2}\right) \frac{dx_2}{dx_3} + \frac{F_f}{l_1} \cos\alpha_2 \cos\alpha_3 \left(1 - \frac{x_1}{l_1} \cdot \frac{dl_1}{dx_1}\right) \frac{dx_1}{dx_3}\Bigg] \tag{S53}$$

$$\Rightarrow \frac{dF_m}{dx_3} = n_1 n_2 n_3 \Bigg[\frac{x_1}{l_1} \cos\alpha_1 \cos\alpha_2 \cos\alpha_3 \frac{dF_f}{d\varepsilon} \cdot \frac{d\varepsilon}{dl_1} \cdot \frac{dx_1}{dx_2} \cdot \frac{dx_2}{dx_3} + \frac{F_f}{l_3} \cos\alpha_1 \cos\alpha_2 \left(1 - \frac{x_3^2}{l_3^2}\right) + \frac{F_f}{l_2} \cos\alpha_3 \cos\alpha_1 \left(1 - \frac{x_2^2}{l_2^2}\right) \frac{dx_2}{dx_3} + \frac{F_f}{l_1} \cos\alpha_2 \cos\alpha_3 \left(1 - \frac{x_1^2}{l_1^2}\right) \frac{dx_1}{dx_3}\Bigg] \tag{S54}$$

Similar to the 2nd level actuator, the geometrical constraint of the displacements of the each arms of the 3rd level actuator are given by is given by:

$$\Delta x_3 = \Delta x_2 \cos\alpha_3 \tag{S55}$$

$$\Delta x_2 = \Delta x_1 \cos\alpha_2 \tag{S56}$$

From equation (S55) and (S56), the stroke (Δx_3) can be expressed as a function of (Δx_1) as follows:

$$\Delta x_3 = \Delta x_1 \cos\alpha_2 \cos\alpha_3 \tag{S57}$$

Upon applying the first principle of derivative on elemental displacement, the following relation can be obtained:

$$\frac{dx_1}{dx_2} = \lim_{\substack{\Delta x_1 \to 0 \\ \Delta x_2 \to 0}} \left(\frac{\Delta x_1}{\Delta x_2}\right) = \sec\alpha_2 \tag{S58}$$

$$\frac{dx_2}{dx_3} = \lim_{\substack{\Delta x_2 \to 0 \\ \Delta x_3 \to 0}} \left(\frac{\Delta x_2}{\Delta x_3}\right) = \sec\alpha_3 \tag{S59}$$

$$\frac{dx_1}{dx_3} = \lim_{\substack{\Delta x_1 \to 0 \\ \Delta x_3 \to 0}} \left(\frac{\Delta x_1}{\Delta x_3}\right) = \sec\alpha_2 \sec\alpha_3 \tag{S60}$$

$$\varepsilon = \frac{l_{10} - l_1}{l_{10}} \Rightarrow \frac{d\varepsilon}{dl_1} = -\frac{1}{l_{10}} \tag{S61}$$

Substituting the values from equations (S58), (S59), (S60) and (S61), the equation (S54) can be modified as:

$$\begin{aligned}\frac{dF_m}{dx_3} = n_1 n_2 n_3 \Bigg[&\cos^2\alpha_1 \cos\alpha_2 \cos\alpha_3 \frac{dF_f}{d\varepsilon}\left(-\frac{1}{l_{10}}\right)\sec\alpha_2 \sec\alpha_3 + \frac{F_f}{l_3}\cos\alpha_1 \cos\alpha_2 \left(1-\cos^2\alpha_3\right) \\ &+ \frac{F_f}{l_2}\cos\alpha_3 \cos\alpha_1 \left(1-\cos^2\alpha_2\right)\sec\alpha_3 + \frac{F_f}{l_1}\cos\alpha_2 \cos\alpha_3 \left(1-\cos^2\alpha_1\right)\sec\alpha_2 \sec\alpha_3\Bigg]\end{aligned} \tag{S62}$$

$$\Rightarrow \frac{dF_m}{dx_3} = n_1 n_2 n_3 \left[-\frac{1}{l_{10}}\cos^2\alpha_1 \frac{dF_f}{d\varepsilon} + \frac{F_f}{l_3}\cos\alpha_1 \cos\alpha_2 \sin^2\alpha_3 + \frac{F_f}{l_2}\cos\alpha_1 \sin^2\alpha_2 + \frac{F_f}{l_1}\sin^2\alpha_1\right] \tag{S63}$$

$$\begin{aligned}\Rightarrow \frac{dF_m}{dx_3} = n_1 n_2 n_3 \Bigg[&-\frac{1}{l_{10}}A_{cross}\cos^2\alpha_1 \frac{d\sigma}{d\varepsilon} + \frac{\sigma}{l_3}A_{cross}\cos\alpha_1 \cos\alpha_2 \sin^2\alpha_3 \\ &+ \frac{\sigma}{l_2}A_{cross}\cos\alpha_1 \sin^2\alpha_2 + \frac{\sigma}{l_{10}(1-\varepsilon)}A_{cross}\sin^2\alpha_1\Bigg]\end{aligned} \tag{S64}$$

where, $F_f = \sigma A_{cross}$, $E = d\sigma/d\varepsilon$, σ is the stress induced in the SMA wire, $l_1 = l_{10}(1-\varepsilon)$ and A_{cross} is the cross-sectional area of the SMA wire,

$$\therefore \frac{dF_m}{dx_3} = n_1 n_2 n_3 A_{cross}\left[-\frac{E}{l_{10}}\cos^2\alpha_1 + \frac{\sigma}{l_3}\cos\alpha_1 \cos\alpha_2 \sin^2\alpha_3 + \frac{\sigma}{l_2}\cos\alpha_1 \sin^2\alpha_2 + \frac{\sigma}{l_{10}(1-\varepsilon)}\sin^2\alpha_1\right] \tag{S65}$$

Combining equations (S45), (S46) and (S65), it can be written as:

$$k_m \Delta x_3 = n_1 n_2 n_3 \sigma A_{cross}\cos\alpha_1 \cos\alpha_2 \cos\alpha_3 - K_x x_0 \tag{S66}$$

$$\begin{aligned}\Rightarrow n_1 n_2 n_3 A_{cross}\Bigg[&-\frac{E}{l_{10}}\cos^2\alpha_1 + \frac{\sigma}{l_3}\cos\alpha_1 \cos\alpha_2 \sin^2\alpha_3 + \frac{\sigma}{l_2}\cos\alpha_1 \sin^2\alpha_2 \\ &+ \frac{\sigma}{l_{10}(1-\varepsilon)}\sin^2\alpha_1\Bigg]\Delta x_3 = n_1 n_2 n_3 \sigma A_{cross}\cos\alpha_1 \cos\alpha_2 \cos\alpha_3 - K_x x_0\end{aligned} \tag{S67}$$

The total displacement or stroke (Δx_3) of the 3rd level hierarchical actuator as a function of stress induced (σ) and strain developed (ε) in the SMA wire is given by:

$$\Delta x_3 = \frac{n_1 n_2 n_3 \sigma A_{cross} \cos\alpha_1 \cos\alpha_2 \cos\alpha_3 - K_x x_0}{n_1 n_2 n_3 A_{cross} \left[-\frac{E}{l_{10}} \cos^2\alpha_1 + \frac{\sigma}{l_3} \cos\alpha_1 \cos\alpha_2 \sin^2\alpha_3 + \frac{\sigma}{l_2} \cos\alpha_1 \sin^2\alpha_2 + \frac{\sigma}{l_{10}(1-\varepsilon)} \sin^2\alpha_1 \right]} \tag{S68}$$

3.5 Bipennate muscle stiffness equation – $\mathbf{N^{th}}$ hierarchical level actuator

Applying the principle of mathematical induction, by extrapolating the results obtained for 1st, 2nd, and 3rd level actuator, and verifying for the fourth stage actuator, the total displacement or stroke (Δx_N) of the Nth level hierarchical actuator as a function of stress induced (σ) and strain developed (ε) in the SMA wire is obtained as:

$$\Delta x_N = \frac{\prod_{i=1}^{n=N} [n_i F_f \cos\alpha_i] - K_x x_0}{\prod_{i=1}^{n=N} [n_i] A_{cross} \left[-\frac{E}{l_{10}} \cos^2\alpha_1 + \sum_{k=2}^{n=N} \left(\frac{\sigma}{l_k} \sin^2\alpha_k \prod_{j=1}^{k-1} \cos\alpha_j \right) + \frac{\sigma}{l_{10}(1-\varepsilon)} \sin^2\alpha_1 \right]} \tag{S69}$$

4 Application

The bipennate muscle-based shape memory alloy actuator has broad applications ranging from building automation controls to precise drug delivery methods. Being compact, it can also act as a viable alternative to replace DC motor gear-train based conventional actuators.

- **Building automation and controls:** Dampers are used in industrial HVAC systems to regulate airflow. Torque ranging from 2 to 50 Nm is required by the actuators that regulate the damper. Higher torque necessitates a larger actuator, which comes at a cost. Higher torque requires an increase in actuator size with cost implications. Shape memory alloy actuators based on bipennate muscles can be used to achieve the same necessary torque with a smaller footprint and lower cost.

- **Drug delivery/mixing applications:** The developed SMA-based actuators can be utilized to control microvalves for cancer patients' precise medication administration systems. High force production permits drug delivery or mixing of a high viscous fluid with micrometer stroke accuracy, which is a unique feature of bipennate muscle based SMA actuators.

- **Magnetic resource imaging (MRI):** Magnetic resonance imaging (MRI) is particularly sensitive to electromagnetic noise produced by traditional coil-based motors, making it difficult to research neurology. Compared to its conventional equivalent, a bipennate muscle-based shape memory alloy actuator may significantly reduce the electromagnetic fields created. This aids in the reception of unaltered magnetic resonance imaging pictures free of electromagnetic noise, allowing for further analysis and extraction of neurological properties.

- **Variable stiffness muscle:** The current system is developed based on the bipennate muscle, which has variable stiffness. The stiffness value of the hierarchical actuator is determined by the change in SMA wire length, pennation angle, and stress generated during actuation. As a result, the present invention functions as a variable stiffness mechanism, which is advantageous for roboticists and engineers since it includes capabilities like energy storage and better human-interaction safety that are not inherent in typical kinematic linkages.

- **Adaptive robotic prostheses:** The interesting property of the customizable multi-stage hierarchy of the shape memory alloy-based bioinspired muscle design will also encourage researchers in the domain of biomechatronics to develop adaptive robotic prostheses.

- **Antenna reconfiguration**: The lab has developed an SMA based solution to control the shape of parabolic antenna reflector and thus control the antenna foot-print for space application of ISRO [12]. This reconfiguration works on a limited actuation.

5 References

1. Lagoudas, D. C. *Shape memory alloys: modeling and engineering applications* (Springer, 2008).

2. Song, S.-H. *et al.* 35 hz shape memory alloy actuator with bending-twisting mode. *Sci. reports* **6**, 1–13 (2016).

3. Fortune-Business-Insights. Actuators market size, share & covid-19 impact analysis, and regional forecast, 2021-2028. *https://www.fortunebusinessinsights.com/actuators-market-103531* **FBI103531**, 160 (2021).

4. Markets-And-Markets. Actuators market by actuation, type, application & vertical, and region global forecast to 2027. *https://www.marketsandmarkets.com/Market-Reports/global-actuators-market-59465451.html* **AS 2252**, 341 (2021).

5. Yuen, M., Cherian, A., Case, J. C., Seipel, J. & Kramer, R. K. Conformable actuation and sensing with robotic fabric. In *2014 IEEE/RSJ international conference on intelligent robots and systems*, 580–586 (IEEE, 2014).

6. Reynaerts, D. & Van Brussel, H. Design aspects of shape memory actuators. *Mechatronics* **8**, 635–656 (1998).

7. Lee, J.-H., Chung, Y. S. & Rodrigue, H. Long shape memory alloy tendon-based soft robotic actuators and implementation as a soft gripper. *Sci. reports* **9**, 1–12 (2019).

8. Mosley, M., Mavroidis, C. & Pfeiffer, C. Design and dynamics of a shape memory alloy wire bundle actuator. In *Proceedings of the ANS, 8th topical meeting on robotics and remote systems*, 1–14 (Citeseer, 1999).

9. Shin, D. D., Mohanchandra, K. P. & Carman, G. P. Development of hydraulic linear actuator using thin film sma. *Sensors Actuators A: Phys.* **119**, 151–156 (2005).

10. Elahinia, M. H. & Ahmadian, M. An enhanced sma phenomenological model: I. the shortcomings of the existing models. *Smart materials structures* **14**, 1297 (2005).

11. Jenkins, T. & Bryant, M. Pennate actuators: force, contraction and stiffness. *Bioinspiration & biomimetics* **15**, 046005 (2020).

12. Kalra, S., Bhattacharya, B. & Munjal, B. Design of shape memory alloy actuated intelligent parabolic antenna for space applications. *Smart Mater. Struct.* **26**, 095015 (2017).

6 Notation

α	pennation angle	E_A	Young's modulus of Austenite phase
Δx	stroke of the actuator	E_M	Young's modulus of Martensite phase
ΔH	latent heat of SMA wire	F_f	fiber force in SMA wire
ε	strain developed in the SMA wire	F_m	muscle force generated by the actuator
ε_L	residual strain	h_T	convective heat transfer co-efficient
θ_T	thermal expansion factor	k_m	muscle stiffness of the actuator
ξ	martensite volume fraction	K_x	spring constant of Bias spring
ξ_A	martensite fraction reached before cooling	l	length of the branch at any time
ξ_M	martensite fraction reached before heating	l_0	initial length of one unipennate branch of SMA wire
ρ	density of the SMA wire	M'_f	stress-modified martensite phase finish temperature
σ	stress in SMA wire	M'_s	stress-modified martensite phase start temperature
Ω	phase transformation contribution factor	M_f	martensite phase finish temperature
A'_f	stress-modified austenite phase finish temperature	M_s	martensite phase start temperature
A'_s	stress-modified austenite phase start temperature	m_{wire}	mass of SMA wire
A_{cross}	cross-sectional area of the SMA wire	N	number of levels in the hierarchical actuator
A_c	curved surface area of SMA wire	n	number of SMA wires in one bipennate structure
A_f	austenite phase finish temperature	r_A	resistivity of the austenite phase
A_s	austenite phase start temperature	r_M	resistivity of the martensite phase
C_A	effect of stress on austenite temperatures	R_{ohm}	resistance of the SMA wire
C_M	effect of stress on martensite temperatures	T	temperature of SMA wire
c_p	specific heat of SMA wire	T_∞	ambient temperature
d	diameter of the SMA wire	V_{in}	voltage input
E	Young's modulus of the SMA	x_0	initial elongation of the bias spring

7 Appendix: Simulink block subsystems

7.1 Constitutive Model: Simulink subsystem

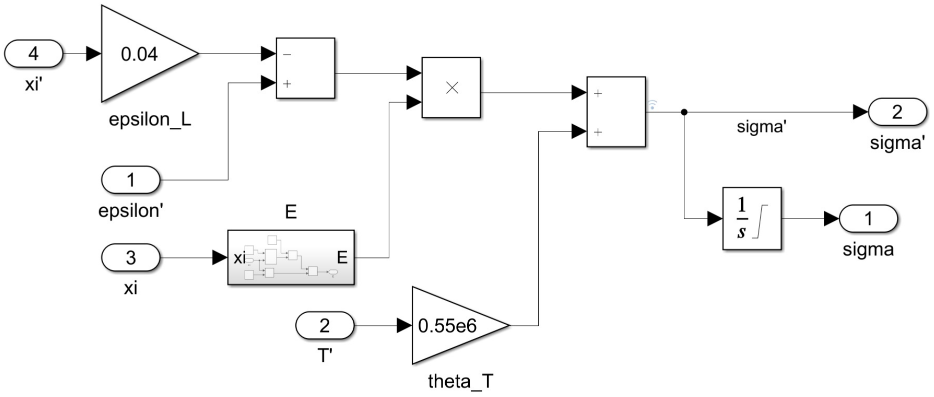

Figure S8. The block diagram depicts the modeling of the constitutive equation relating the SMA stress to the thermomechanical variables such as strain, martensite volume fraction, and temperature, as mentioned in equation (2) (refer to main article file). The subsystem gives the output in terms of the stress rate of each SMA wire present in the bipennate-based SMA actuator.

7.2 Heat Transfer Model: Simulink subsystem

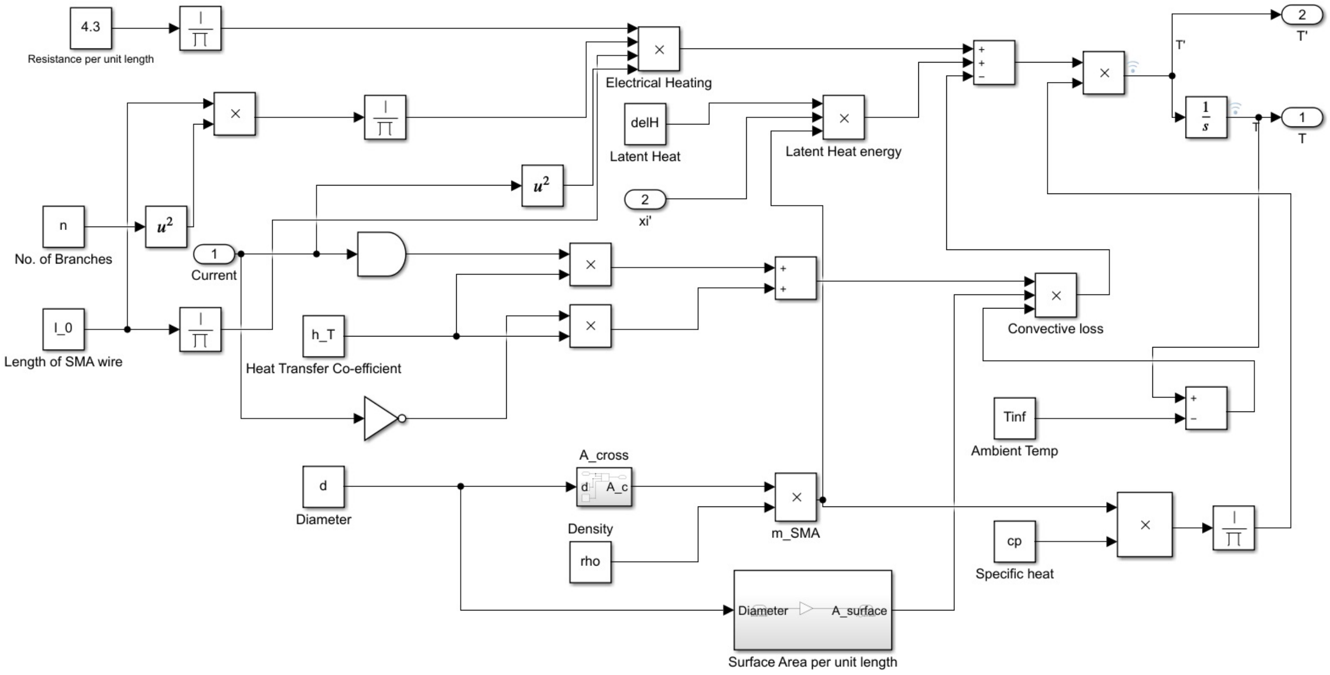

Figure S9. The block diagram illustrates the heat energy balance equation (6) (refer to main article file). The heat energy required to increase the SMA wire temperature is obtained through the Joule heating effect. The output of the subsystem is in terms of the temperature rate of each SMA wire present in the bipennate-based SMA actuator.

7.3 Phase Transformation Model: Simulink subsystem

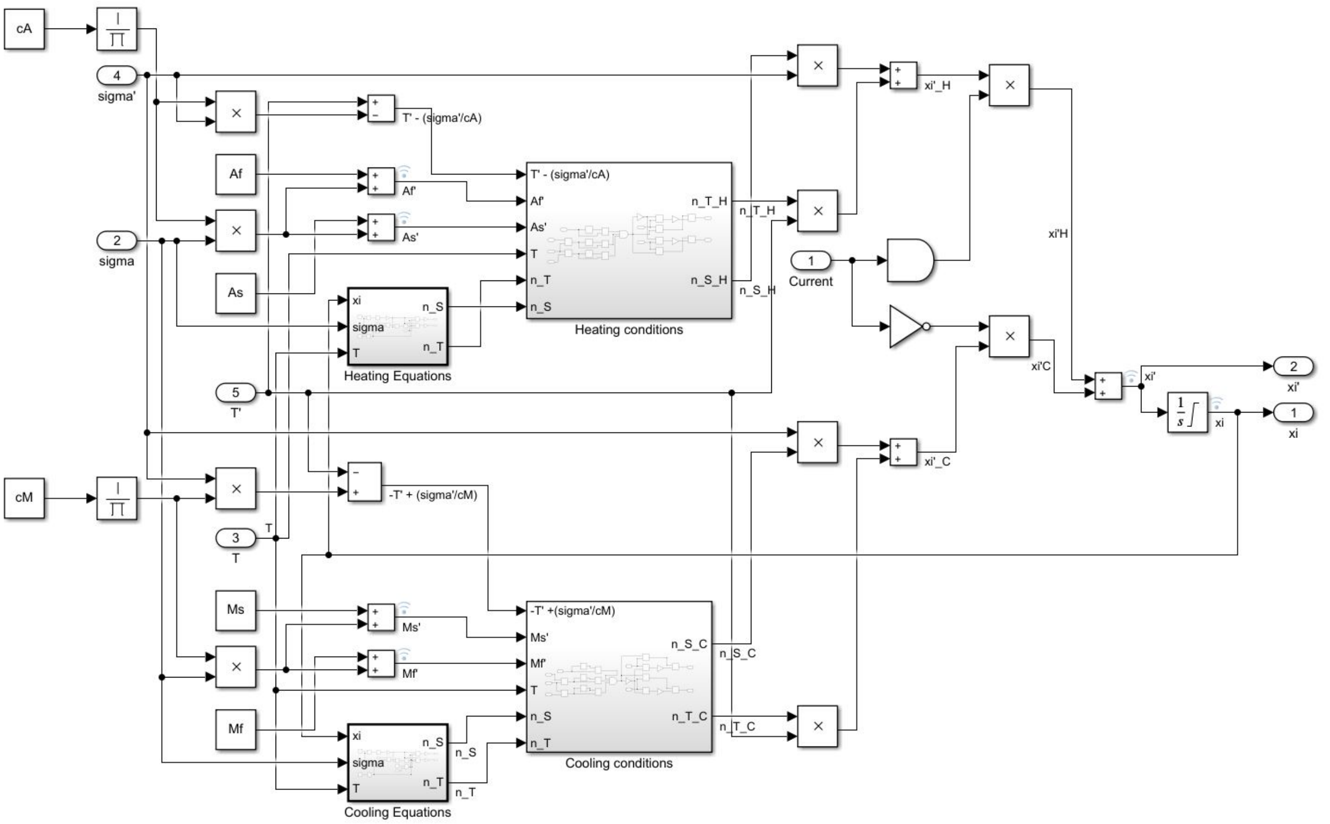

Figure S10. The block diagram depicts the enhanced phenomenological phase transformation model. The subsystem incorporates both the cases of forward and reverse phase transformations. The output of the subsystem is in terms of martensite volume fraction as represented in equations (3)-(5) (refer to main article file).

7.3.1 Heating Equations: Simulink sub-model

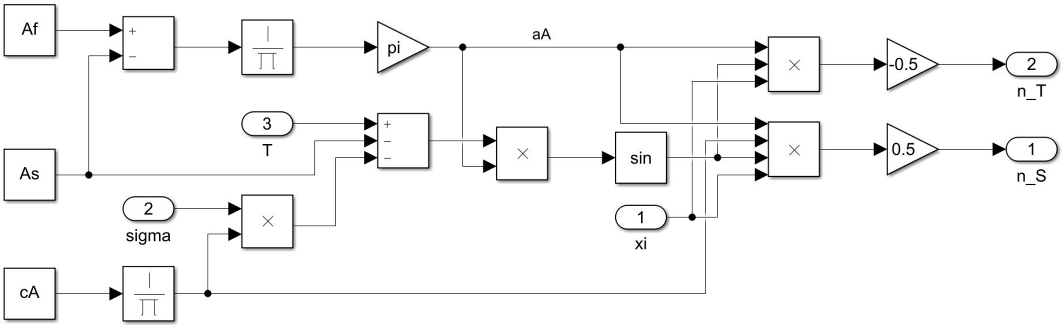

Figure S11. The block diagram depicts the subsystem to the phase transformation model, which formulates the constant to the enhanced phenomenological model during the reverse transformation as represented in equation (3) (refer to main article file).

7.3.2 Heating Conditions: Simulink sub-model

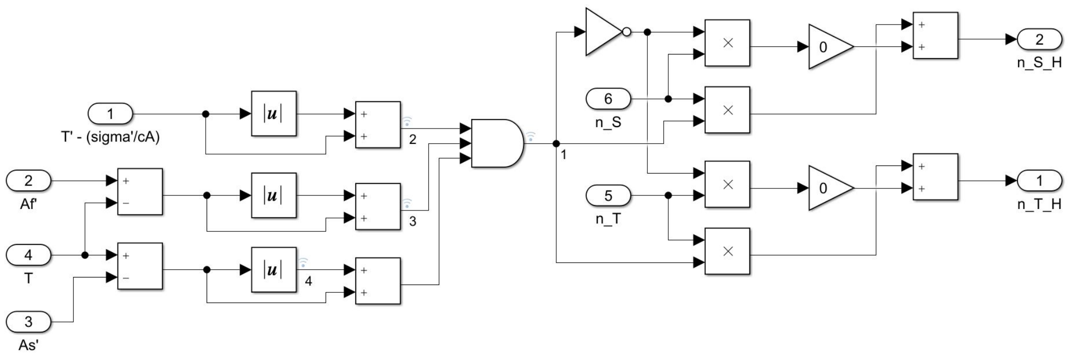

Figure S12. The block diagram depicts the subsystem to the phase transformation model, which considers three different cases during the reverse transformation, (a) $T < A'_s$ (b) $A'_s < T < A'_f$ (c) $T > A'_f$, where, A'_s is the stress modified austenite start temperature and, A'_f denotes the stress modified austenite finish temperature.

7.3.3 Cooling Equations: Simulink sub-model

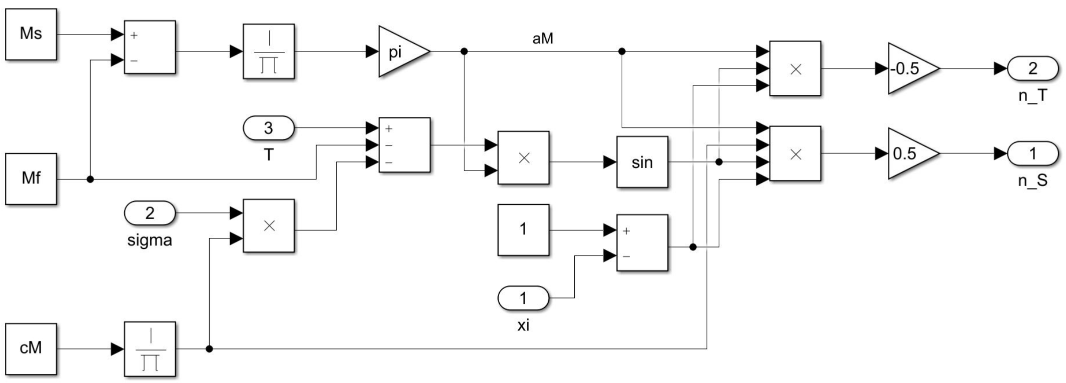

Figure S13. The block diagram depicts the subsystem to the phase transformation model, which formulates the constant to the enhanced phenomenological model during the forward transformation as represented in equation (4) (refer to main article file).

7.3.4 Cooling Conditions: Simulink sub-model

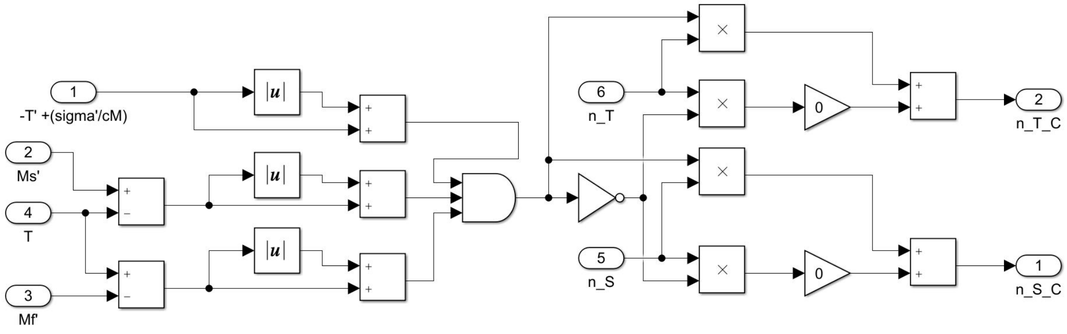

Figure S14. The block diagram depicts the subsystem to the phase transformation model, which considers three different cases during the forward transformation, (a) $T > M'_s$ (b) $M'_f < T < M'_s$ (c) $T < M'_f$, where, M'_s is the stress modified martensite start temperature and, M'_f denotes the stress modified martensite finish temperature.

7.4 Dynamics Model: Simulink subsystem

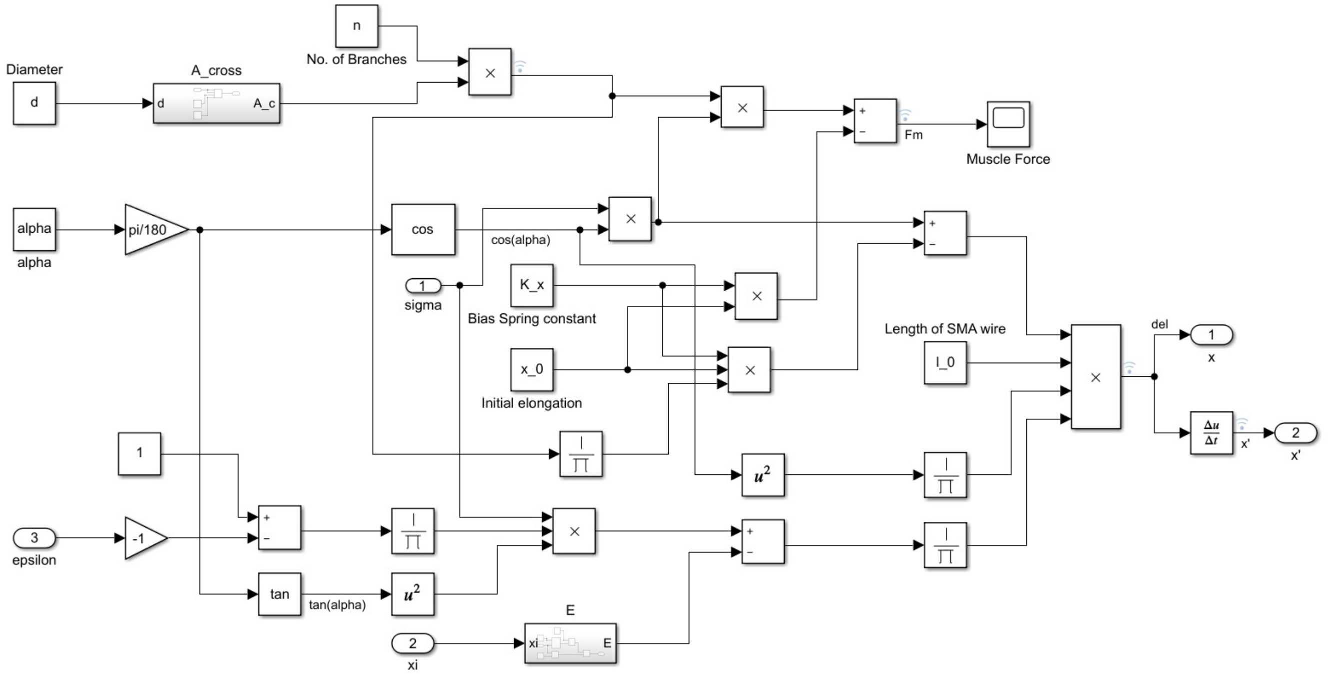

Figure S15. The block diagram depicts the dynamics model subsystem of the mathematical model represented by equation (10) (refer to main article file). The output of the subsystem is in terms of the stroke for the bipennate-based SMA actuator.

7.5 Kinematics Model: Simulink subsystem

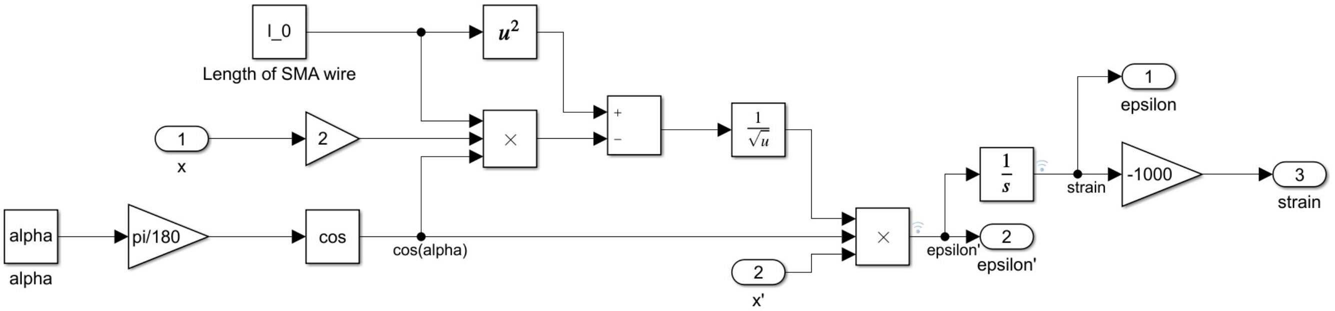

Figure S16. The block diagram depicts the kinematics model subsystem of the mathematical model represented by equation (12) (refer to main article file). The output of the subsystem is in terms of the strain rate observed in each SMA wire present in the bipennate-based SMA actuator.
